# Leveraging transcription factors to speed cellobiose fermentation by *Saccharomyces cerevisiae*

**DOI:** 10.1186/s13068-014-0126-6

**Published:** 2014-08-27

**Authors:** Yuping Lin, Kulika Chomvong, Ligia Acosta-Sampson, Raíssa Estrela, Jonathan M Galazka, Soo Rin Kim, Yong-Su Jin, Jamie HD Cate

**Affiliations:** Departments of Molecular and Cell Biology, University of California, Berkeley, CA 94720 USA; Plant and Microbial Biology, University of California, Berkeley, CA 94720 USA; Chemistry, University of California, Berkeley, CA 94720 USA; Physical Biosciences Division, Lawrence Berkeley National Laboratory, Berkeley, CA 94720 USA; Department of Food Science and Human Nutrition, University of Illinois at Urbana-Champaign, Urbana, Illinois 61801 USA; Institute for Genomic Biology, University of Illinois at Urbana-Champaign, Urbana, Illinois 61801 USA

**Keywords:** Cellobiose, Glycolysis, Systems biology, Transcription factor, Metabolic engineering, Biofuels

## Abstract

**Background:**

*Saccharomyces cerevisiae*, a key organism used for the manufacture of renewable fuels and chemicals, has been engineered to utilize non-native sugars derived from plant cell walls, such as cellobiose and xylose. However, the rates and efficiencies of these non-native sugar fermentations pale in comparison with those of glucose. Systems biology methods, used to understand biological networks, hold promise for rational microbial strain development in metabolic engineering. Here, we present a systematic strategy for optimizing non-native sugar fermentation by recombinant *S. cerevisiae*, using cellobiose as a model.

**Results:**

Differences in gene expression between cellobiose and glucose metabolism revealed by RNA deep sequencing indicated that cellobiose metabolism induces mitochondrial activation and reduces amino acid biosynthesis under fermentation conditions. Furthermore, glucose-sensing and signaling pathways and their target genes, including the cAMP-dependent protein kinase A pathway controlling the majority of glucose-induced changes, the Snf3-Rgt2-Rgt1 pathway regulating hexose transport, and the Snf1-Mig1 glucose repression pathway, were at most only partially activated under cellobiose conditions. To separate correlations from causative effects, the expression levels of 19 transcription factors perturbed under cellobiose conditions were modulated, and the three strongest promoters under cellobiose conditions were applied to fine-tune expression of the heterologous cellobiose-utilizing pathway. Of the changes in these 19 transcription factors, only overexpression of *SUT1* or deletion of *HAP4* consistently improved cellobiose fermentation. *SUT1* overexpression and *HAP4* deletion were not synergistic, suggesting that *SUT1* and *HAP4* may regulate overlapping genes important for improved cellobiose fermentation. Transcription factor modulation coupled with rational tuning of the cellobiose consumption pathway significantly improved cellobiose fermentation.

**Conclusions:**

We used systems-level input to reveal the regulatory mechanisms underlying suboptimal metabolism of the non-glucose sugar cellobiose. By identifying key transcription factors that cause suboptimal cellobiose fermentation in engineered *S. cerevisiae*, and by fine-tuning the expression of a heterologous cellobiose consumption pathway, we were able to greatly improve cellobiose fermentation by engineered *S. cerevisiae*. Our results demonstrate a powerful strategy for applying systems biology methods to rapidly identify metabolic engineering targets and overcome bottlenecks in performance of engineered strains.

**Electronic supplementary material:**

The online version of this article (doi:10.1186/s13068-014-0126-6) contains supplementary material, which is available to authorized users.

## Background

**Figure 1:**
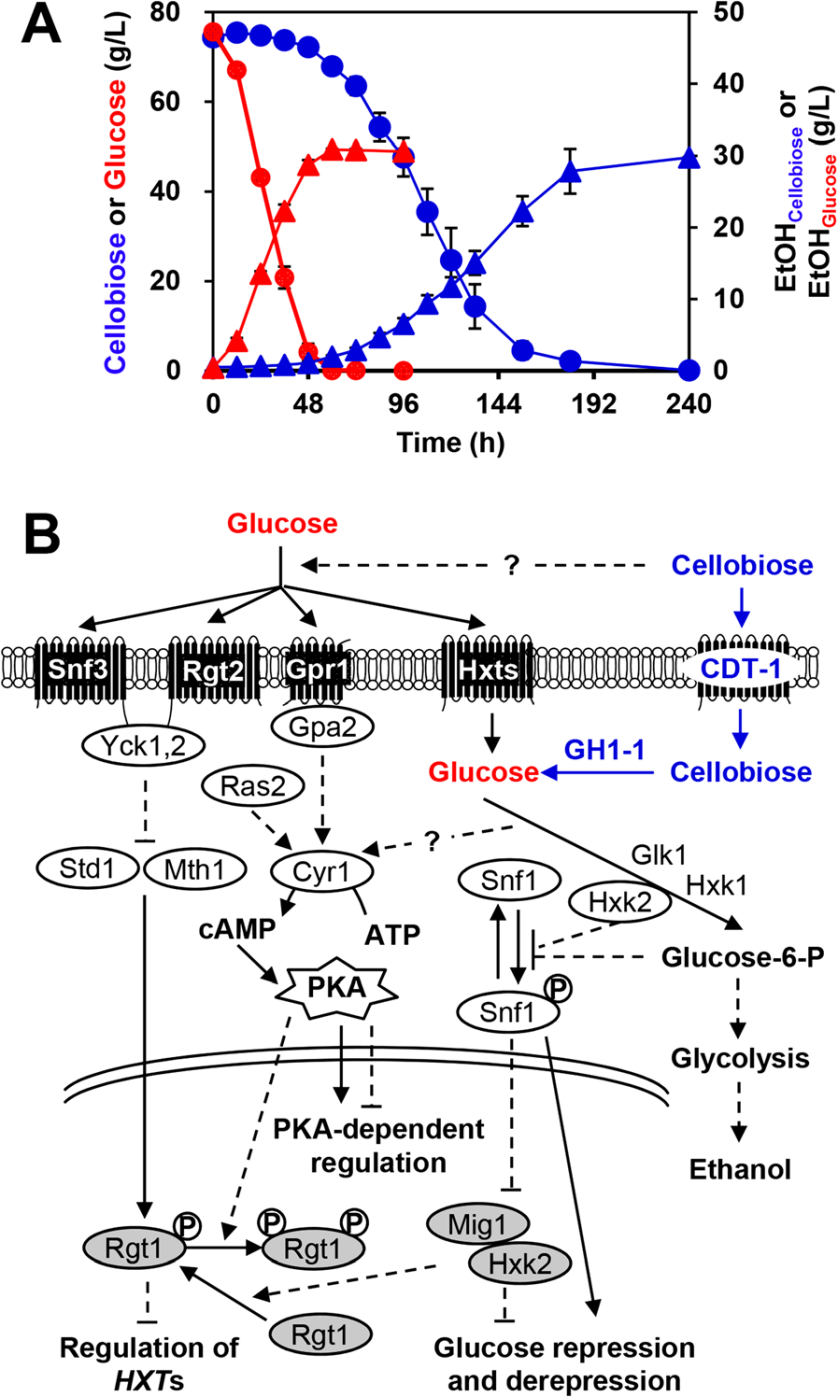
**Suboptimal cellobiose metabolism in engineered** ***Saccharomyces cerevisiae*** **. (A)** Fermentation profiles of recombinant cellobiose-utilizing *S. cerevisiae* with plasmid pRS426-BT on cellobiose or glucose in anaerobic conditions with an initial OD_600_ of 1. Concentrations: cellobiose (blue circle), glucose (red circle), ethanol from cellobiose (blue triangle), and ethanol from glucose (red triangle). Data represent the mean and standard error of triplicate cultures grown on each source. The arrows indicate the times at which samples were taken for transcription profiling by RNA deep sequencing. **(B)** Model of the regulation of glucose metabolism and of glucose-sensing and signaling networks in the context of a cellobiose-utilizing pathway. The cellobiose-utilizing pathway was established in *S. cerevisiae* by introducing a cellodextrin transporter gene (*cdt-1*) and an intracellular β-glucosidase gene (*gh1-1*) from *Neurospora crassa*. Gpr1 and Gpa2 define a glucose-sensing pathway that works in parallel with Ras2 to activate protein kinase A (PKA), which induces genome-wide regulation. Signals emanating from Snf3 and Rgt2 regulate hexose transporter genes by inactivating the Rgt1 co-repressors Mth1 and Std1. The glucose repression signal that inactivates Snf1 kinase is generated through glucose metabolism, consequently inducing the Mig1/Hxk2-mediated glucose repression pathway. In addition, Snf1 kinase directly mediates phosphorylation of transcription activators of glucose-repressed genes to relieve glucose repression.

*Saccharomyces cerevisiae* is a key organism used for the manufacture of renewable fuels and chemicals, but it is not capable of using mixed sugars derived from the plant cell wall [1–4]. Efforts to broaden the substrate spectrum of *S. cerevisiae* beyond glucose include the use of an intracellular cellobiose-degrading pathway composed of a cellodextrin transporter and an intracellular β-glucosidase [5]. Using this approach, *S. cerevisiae* has been engineered to co-ferment cellobiose and xylose [6] or cellobiose and galactose [7]. However, the rates and efficiencies of cellobiose, xylose, and other non-glucose sugar fermentations pale in comparison with those of glucose [8–10], hampering the application of non-glucose fermentation on an industrial scale. For example, suboptimal cellobiose metabolism results in prolonged lag phases and slow rates, albeit with similar ethanol yields, compared with glucose metabolism (Figure 1A) [5,6,11–14].

*S. cerevisiae* has evolved hierarchical gene regulatory networks (GRNs) that respond to glucose, and these allow glucose to be consumed rapidly and preferentially even when non-glucose sugars are present [15–18] (Figure 1B). GRNs controlling the preferential use of glucose are also prevalent in bacteria and other eukaryotes [19,20].

In *S. cerevisiae*, three glucose-sensing and signaling pathways have been identified. The first depends on the cAMP-dependent protein kinase A (PKA), which is activated by the G protein-coupled receptor (Gpr1/Gpa2) and Ras2, which in parallel control the majority of glucose-induced changes in gene expression via modulating transcription factors (TFs) [21]. In the second, two transmembrane sensors of extracellular glucose, Snf3 and Rgt2, converge on the Rgt1 repressor to regulate the expression of glucose transporters [22]. The third main glucose repression pathway involves the Snf1 kinase complex, which inhibits the Mig1 repressor-containing complex, thereby repressing genes involved in respiration, gluconeogenesis, and the metabolism of alternative carbon sources [23]. These three systems operate as interconnected GRNs. For example, Rgt1 function is mainly altered by the signals generated by Snf3 and Rgt2, but is also regulated by the PKA and Snf1-Mig1 glucose repression pathways [24].

The TFs in the *S. cerevisiae* glucose-sensing pathways play a central role in layered regulatory networks through complex, combinatorial functions on promoters that are not fully understood [25,26], and that may not be conserved across *S. cerevisiae* species [27,28]. By contrast, cellobiose is an unusual substrate for *S. cerevisiae*, and is therefore not recognized as a readily fermentable sugar like glucose. Efforts to optimize cellobiose fermentation in engineered *S. cerevisiae* through combinatorial transcriptional engineering [11], experimental evolution [29], or by exploring and evolving new cellodextrin transporters [12,14,30,31] or an alternative cellobiose phosphorylase pathway [29,32,33] have resulted in only limited improvements in cellobiose metabolism.

To improve non-native sugar metabolism in *S. cerevisiae*, systems-level measurements could be exploited to provide important insights into unknown bottlenecks present in engineered strains exhibiting suboptimal performance. Here, we present a strategy for optimizing non-native sugar fermentation by recombinant *S. cerevisiae* using cellobiose as a model. First, the GRNs perturbed by cellobiose were identified by RNA deep sequencing of the transcriptomes of engineered *S. cerevisiae* growing on cellobiose or glucose. Second, to identify the underlying causes of suboptimal cellobiose consumption, TFs with significant changes in expression between cellobiose and glucose metabolism in the systems-level analysis were perturbed by deletion or overexpression to examine their effect on cellobiose fermentation. Third, promoters with differing strengths under cellobiose conditions were identified using the transcription profiling data, and were used to fine-tune the expression of the cellobiose-utilizing pathway. Issues of local and global optimizations for strain improvement are longstanding in metabolic engineering, but clear methodologies for these optimizations are not well developed. By harnessing systems-level experiments, we identified key TFs that cause suboptimal cellobiose fermentation, and fine-tuned the expression of the heterologous cellobiose consumption pathway to greatly improve cellobiose fermentation by engineered *S. cerevisiae*. Our results reveal regulatory mechanisms underlying suboptimal non-glucose sugar fermentation in yeast, and demonstrate a promising paradigm for systems biology-based rational microbial strain engineering.

## Results

### Transcriptional reprogramming in response to cellobiose

**Figure 2:**
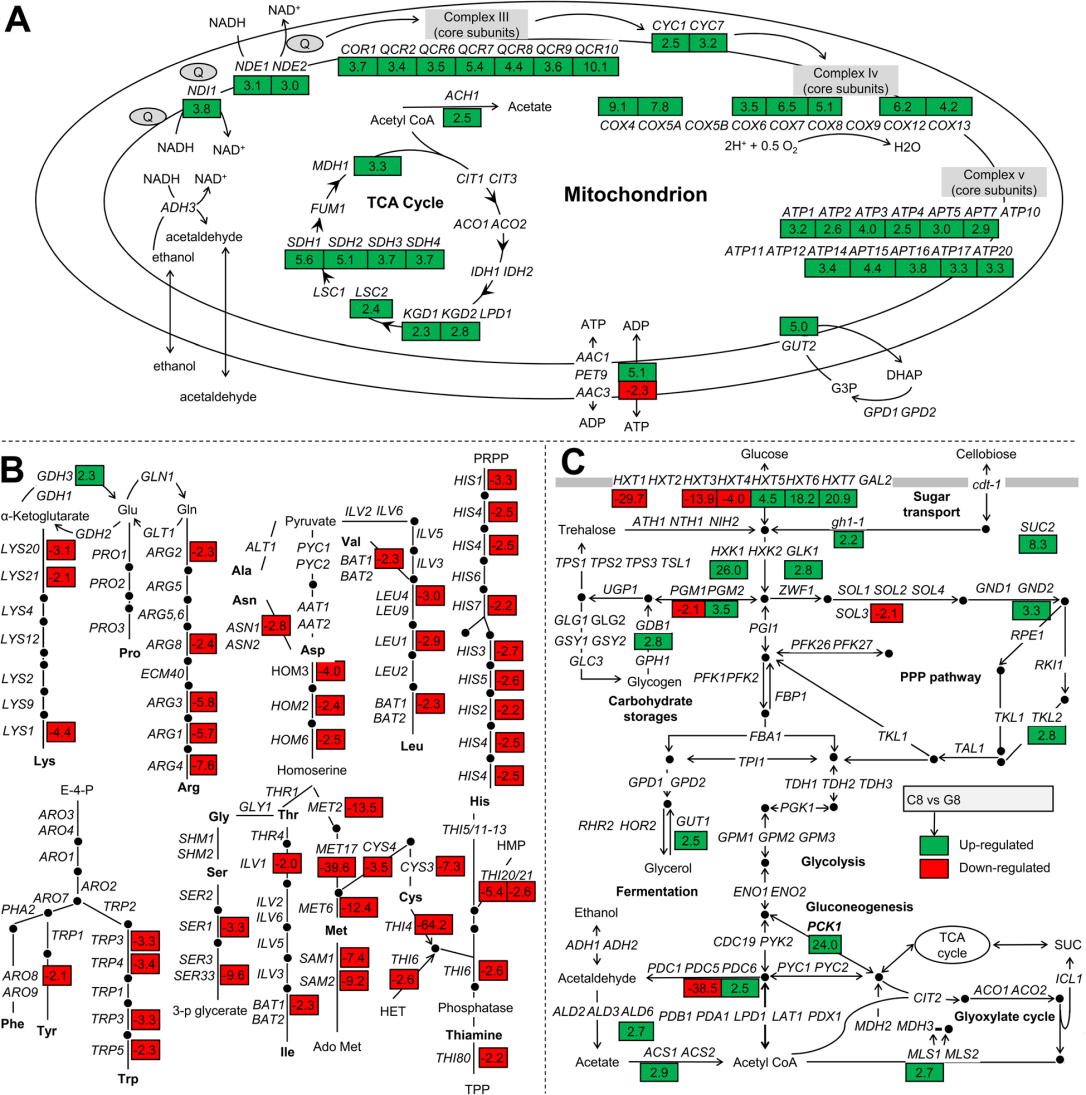
**Impact of cellobiose on central carbon metabolism, amino acid biosynthesis and thiamine biosynthesis of** ***Saccharomyces cerevisiae*** **. (A)** Genes involved in mitochondrial function, including the tricarboxylic acid (TCA) cycle, electron transport chain, and oxidative phosphorylation are shown. **(B)** Genes involved in amino acid and thiamine biosynthesis. **(C)** Genes involved in sugar transport, glycolysis and fermentation, the pentose phosphate pathway, gluconeogenesis, storage of carbohydrates (trehalose and glycogen), and the glyoxylate cycle. **(A–C)** Only genes with significantly different expression when comparing cellobiose-grown versus glucose-grown cells are shown (color-coded boxes), including the fold change on cellobiose (C8) versus glucose (G8). Transcription levels that significantly increased or decreased on cellobiose in contrast to glucose (absolute fold changes ≥2.0, *P* ≤ 0.001) are shown in green and red boxes, respectively.

*S. cerevisiae* cannot metabolize cellobiose naturally. For this study, *S. cerevisiae* was engineered to achieve cellobiose utilization by introducing both a cellodextrin transporter gene (*cdt-1*) and an intracellular β-glucosidase gene (*gh1-1*) from *Neurospora crassa* (See Methods). Although cellobiose is a dimer of glucose, metabolism of cellobiose by engineered *S. cerevisiae* shows substantially prolonged lag phases and slow rates compared with glucose metabolism (Figure 1A). To probe the transcriptional regulatory response of *S. cerevisiae* to cellobiose, we quantified mRNA abundance during exponential growth on either cellobiose or glucose under anaerobic conditions. Transcription profiling (see Additional file 1: Dataset S1) revealed that 519 (8.2%) of the 6,351 genes annotated in the *S. cerevisiae* genome had significantly different expression in cellobiose-grown cells compared with glucose-grown cells (absolute fold changes ≥ 2.0 or more; *P* ≤ 0.001) (see Additional file 2: Dataset S2). Based on Gene Ontology (GO) biological process enrichment analysis (see Additional file 3: Dataset S3), genes with significantly increased mRNA levels (fold changes ≥ 2.0, *P* ≤ 0.001) on cellobiose were enriched for mitochondria-associated processes (Figure 2A), such as ATP biosynthesis, mitochondrial electron and proton transport, and the tricarboxylic acid cycle. Genes with significantly decreased mRNA levels (fold changes ≥ 2.0, *P* ≤ 0.001) on cellobiose were enriched for amino acid (mainly methionine, cysteine, arginine, and histidine) and thiamine (vitamin B1) biosynthetic processes (Figure 2B).

Only a few genes in the pathways of central carbon metabolism [34], which include the pentose phosphate pathway, glyoxylate cycle, gluconeogenesis, and carbohydrate storage (trehalose and glycogen), changed expression levels in the presence of cellobiose compared with glucose (Figure 2C), even though cellobiose fermentation was much slower than glucose fermentation (Figure 1A). The transcript levels of the three glucose-phosphorylating enzymes, encoded by *HXK1*, *HXK2*, and *GLK1*, shifted to the profile similar to that of non-fermentable carbon sources in the cellobiose-grown cells [35] (Figure 2C). When yeast cells are grown on a fermentable carbon source such as glucose, fructose, or mannose, *HXK2* is induced [35]. After shifting cells to a non-fermentable carbon source such as ethanol, *HXK2* is repressed, and *HXK1* and *GLK1* are immediately de-repressed. Cells grown on cellobiose induced the expression of *HXK1* and *GLK1* by 26-fold and 2.8-fold, respectively, over that in glucose-grown cells, although the mRNA levels of *HXK2* were unchanged. Hxk1 seemed to be the predominant isoenzyme in cellobiose-grown cells, because *HXK1* was highly transcribed in contrast to the other two hexokinase genes (see Additional file 1: Dataset S1). Other genes in central carbon metabolic pathways with large changes in expression included the genes coding for hexose transporters, a key gluconeogenic gene (*PCK1*), and genes in mitochondrially compartmentalized pathways (Figure 2A, C). *S. cerevisiae* senses glucose both intracellularly and extracellularly over a wide range of concentrations, which possibly explains the shifts in hexose transporter expression levels observed here, which would be due to the lack of extracellular glucose in the cellobiose cultures [22].

### Effects of TF modulation on cellobiose fermentation

**Figure 3:**
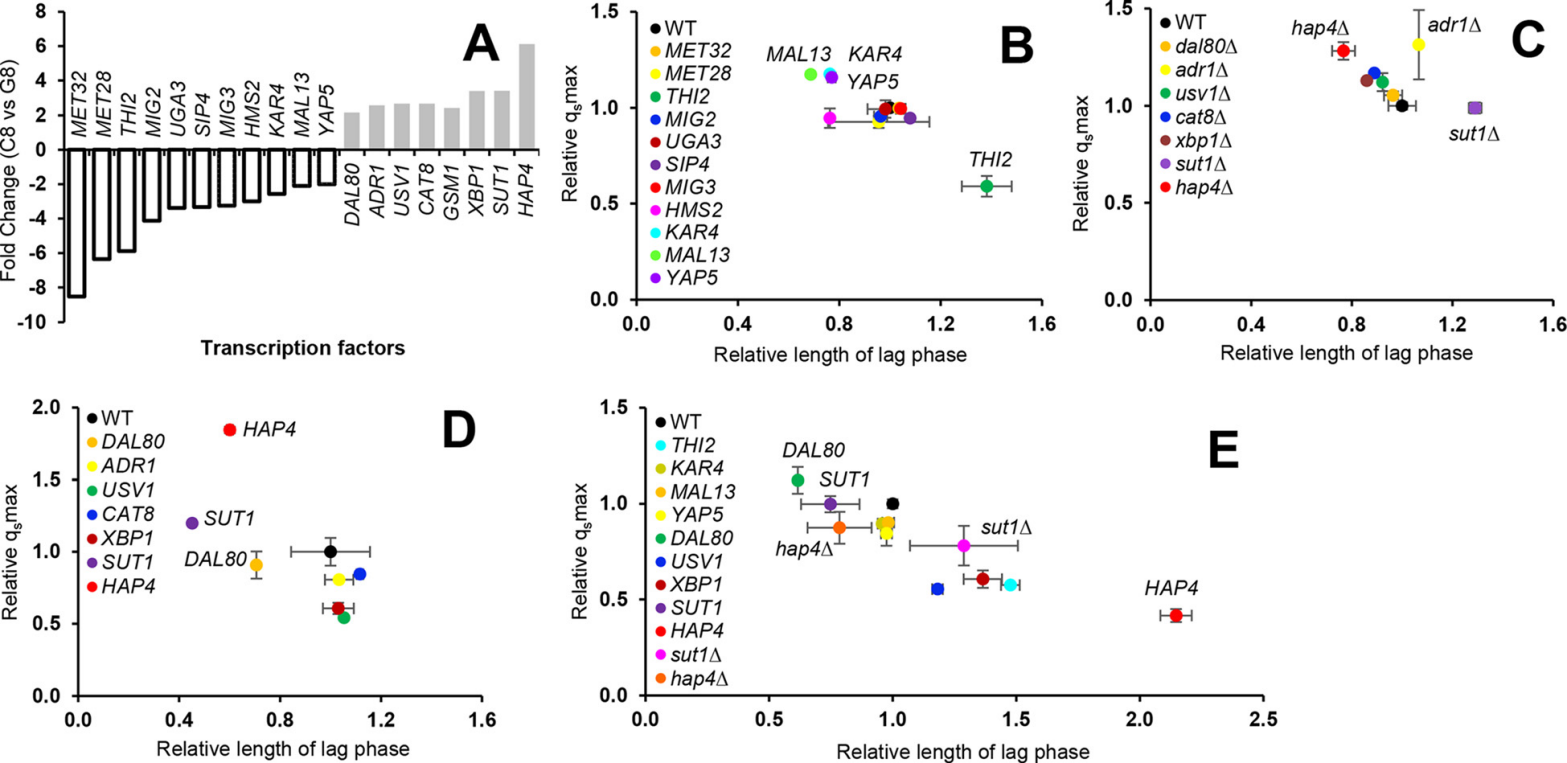
**The effect of manipulating transcription factors (TFs) on cellobiose fermentation. (A)** TFs with significantly different expression levels on cellobiose versus glucose. For all TFs shown, *P*-values were well below 0.001 (see Additional file 2: Dataset S2). **(B)** Cellobiose fermentation profiles of TFs overexpressed in strain BY4742. The TFs chosen for overexpression were those downregulated in cellobiose in the wild-type (WT) strain. The relative cellobiose-consumption rate (q_s_max) and the relative length of the lag phase [100,101] were obtained by comparisons WT controls (normalized to 1.0). **(C)** Cellobiose fermentation profiles of deletion strains in BY4742 background for TFs upregulated in cellobiose versus glucose in **(A)**. **(D)** Cellobiose fermentation profiles of strains overexpressing TFs upregulated on cellobiose versus glucose in **(A)**. **(E)** Cellobiose fermentation profiles using TF mutants in strain D452-2. Because D452-2, which is another laboratory strain of *S. cerevisiae*, seems to have comparable fermentation performance to industrial strains, this strain has attracted attention as a host strain to express foreign sugar-utilizing pathways. In all of the above panels, the cellobiose-consumption pathway was expressed from plasmid pRS316-BT. For panels **(B-E)**, plots of relative q_s_max versus relative length of the lag time are shown. Each point represents duplicate anaerobic fermentations using a starting cellobiose concentration of 80 g/l and starting OD_600_ of 1. The maximum cellobiose-consumption rate and length of the lag phase were 1.22 ± 0.09 g/l/h and 74.28 ± 3.70 h, respectively, for the WT BY4742 strain, and 1.18 ± 0.00 g/l/h and 58.32 ± 2.77 h for the WT D452-2 strain.

As a first step to uncover the regulatory mechanisms underlying suboptimal cellobiose metabolism and thereby to improve cellobiose fermentation by engineered *S. cerevisiae*, TFs were targeted because of their central position in GRNs [25,26]. Of the genes with significantly different expression on cellobiose versus glucose, only 19 annotated TFs were identified (Figure 3A; see Additional file 2: Dataset S2). These 19 TFs are widely distributed in the regulatory network of genes for diverse biological processes, many of which with no obvious connection to central carbon metabolism. Of the 11 TFs with decreased expression on cellobiose, Met32 and Met28 are transcription activators of genes in sulfur-containing amino acid metabolism [36]; Thi2 is an activator of thiamine biosynthetic genes [37]; Yap5 is an iron-responsive activator involved in the diauxic shift from glycolysis to aerobic utilization of ethanol [38,39]; and Uga3 [40], Hms2 [41], and Kar4 [42] are not directly involved in gene regulation of central carbon metabolism. TFs that do have a connection to central carbon metabolism include Mig2 and Mig3, which cooperate with Mig1 to repress many genes in glucose-induced repression [43]; Sip4, an activator of gluconeogenic genes that is regulated by Snf1 [44]; and Mal13, an activator for genes in maltose utilization [45]. Of the eight TFs with increased expression on cellobiose, Dal80 is a negative regulator of genes subject to nitrogen catabolite repression (NCR) in nitrogen degradation pathways [46,47]; Sut1 positively regulates genes in sterol uptake under anaerobic conditions and hypoxic gene expression [48,49]; Xbp1 is involved in gene regulation during stress or starvation [50]; and Gsm1 is thought to be involved in energy metabolism [51]. TFs related to central carbon metabolism include Usv1, which regulates genes during growth on non-fermentable carbon sources [52]; Adr1, an activator of glucose repressible genes that is positively regulated by Snf1 [53,54]; Cat8, which mediates derepression of many genes during the diauxic shift, and which is upregulated by Snf1 through inactivation of the repressor Mig1 as well as being activated directly by phosphorylation [55,56]; and Hap4, which is an activator of respiratory genes and mitochondrial function [57,58].

To determine the functions of these TFs in GRNs relevant to cellobiose metabolism, the effect of their genetic perturbation on cellobiose fermentation was evaluated. For the 11 TFs with decreased expression on cellobiose, overexpression mutants were tested to determine if suboptimal cellobiose fermentation might be due to their decreased expression on cellobiose (Figure 3B; see Additional file 4: Figure S1A). Most of these mutants showed similar lag phases and fermentation rates on cellobiose. Overexpression of *KAR4*, *MAL13*, or *YAP5* in strain BY4742 was slightly beneficial to cellobiose fermentation, whereas *THI2* overexpression strongly repressed cellobiose fermentation. To probe the connection between the eight TFs induced under cellobiose growth conditions and suboptimal cellobiose fermentation, both deletion and overexpression mutants were tested. As with the 11 TFs above, most deletion or overexpressing strains showed similar lag times and fermentation rates on cellobiose compared with wild-type (WT) yeast (Figure 3C, D; see Additional file 4: Figure S1B, C). The *HAP4* deletion strain exhibited shorter lag phases and higher fermentation rates on cellobiose compared with the WT, whereas the *ADR1* deletion strain had an increased cellobiose-consumption rate, but had no change in the lag phase and ethanol productivity. The *SUT1* deletion strain showed clearly longer lag phases on cellobiose compared with the WT BY4742 strain. When each of the eight TFs was overexpressed individually, *SUT1* increased fermentation rates over that of the WT strain, and also reduced the lag phase significantly. Interestingly, overexpression of *HAP4*, of which deletion led to improved cellobiose fermentation, also showed a shorter lag period and a higher cellobiose fermentation rate compared with the control strain. This result suggests that the functions of Hap4 in GRNs might not be monotonic. *DAL80* overexpression reduced the lag phase on cellobiose, but fermentation rates were similar to the WT.

*S. cerevisiae* GRNs have been shown to be strain-dependent [27,28], as has the promoter dependence of the cellobiose-consumption pathway [11]. Therefore, TF expression levels that elicited significant differences in cellobiose fermentation compared with WT in the BY4742 background were further evaluated in the D452-2 (*MATα*, *leu2*, *his3*, *ura3*, and *can1*) strain background [59] (Figure 3E; see Additional file 4: Figure S1D). Testing of the TFs showed that overexpression of *KAR4*, *MAL13*, *YAP5*, or *HAP4* had different effects on cellobiose fermentation in the two strain backgrounds. Strikingly, *HAP4* overexpression, which greatly enhanced cellobiose utilization in BY4742, had a strongly negative impact in D452-2 (Figure 3D, E). Again, this result suggests that the function of Hap4 in GRNs might not be monotonic, so that subtle expression level changes of Hap4 in the context of global regulation of other TFs in GRNs could lead to inconsistent phenotypes depending on the host strain backgrounds. Taken together, the results showed that only three TF mutations resulted in similar phenotypes in the two tested strains: *SUT1* overexpression, *HAP4* deletion, and *DAL80* overexpression (Figure 3E; see Additional file 4: Figure S1D).

Industrial application of *S. cerevisiae* to plant biomass conversion will probably require co-consumption of multiple sugars derived from the plant cell wall, such as cellobiose and xylose [2,13]. To test whether *SUT1* or *DAL80* overexpression would affect xylose fermentation, a uracil auxotroph of the SR8 strain, which is an engineered and evolved xylose-fermenting strain derived from D452-2 [60], was used. SR8 expresses *XYL1* (xylose reductase gene), *XYL2* (xylitol dehydrogenase gene), and *XKS1* (xylulose kinase gene) from chromosomally integrated copies. Further, *ALD6* (aldehyde dehydrogenase gene) was deleted, resulting in strain SR8, which was comparable with the best-performing engineered *S. cerevisiae* strains reported for xylose fermentation [61]. Overexpression of *SUT1* or *DAL80* had no impact on xylose fermentation compared with the empty vector control (see Additional file 4: Figure S2). These results suggest that the reprogramming of GRNs through *SUT1* and *DAL80* overexpression did not affect expression levels of enzymes in the biochemical route for xylose metabolism [62].

### Optimization of the heterologous cellobiose-consumption pathway through promoter engineering

**Figure 4:**
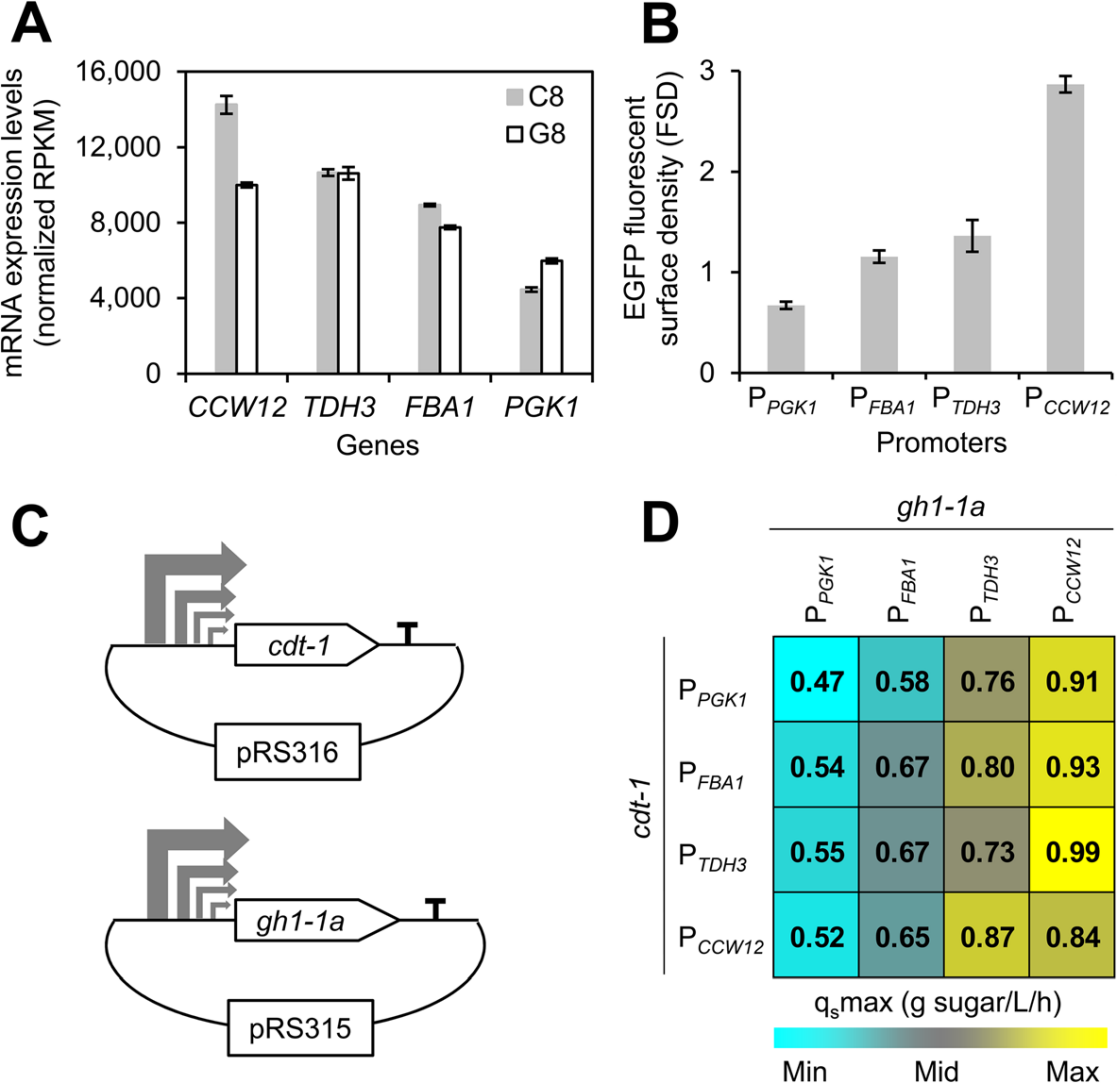
**Promoter engineering to optimize expression of the heterologous cellobiose-utilizing pathway. (A)** Identification of strong promoters from transcription profiling of cellobiose-grown (C8) and glucose-grown (G8) cultures. **(B)** Verification of promoter strengths by measuring green fluorescent protein (GFP) fluorescence using flow cytometry. Anaerobically grown cells on cellobiose were harvested at mid-exponential phase and analyzed. The cell surface density of enhanced green fluorescent protein (eGFP)-tagged CDT-1 is shown. **(C)** Construction of a promoter library of 4 promoters and 2 gesnes for expression of the cellobiose-utilization pathway. The plasmids pRS316 (*CEN URA*) and pRS315 (*CEN LEU*) were used to express *cdt-1* and a codon-optimized version of *N. crassa gh1-1* (*gh1-1a*), respectively. **(D)** Comparison of cellobiose-consumption rates (q_s_max) using strains expressing the cellobiose-utilization pathway from the promoter library. The starting OD_600_ of 1 was used. The promoters for each gene are shown, and the rates are color-coded by relative rates. Fermentation parameters were calculated from the fermentation profiles in Fig. S4*D*.

In addition to reprogramming of GRNs via perturbations of TFs for improved cellobiose fermentation, expression levels of the heterologous cellobiose-assimilation pathway should be optimized to achieve efficient and rapid fermentation of cellobiose. Promoter engineering has been widely and successfully applied to optimize foreign pathways in host strains [63]. Recently, an approach named “customized optimization of metabolic pathways by combinatorial transcriptional engineering” (COMPACTER) was successfully applied to improve recombinant cellobiose-fermenting *S. cerevisiae* stains by fine-tuning gene expression in a cellobiose-utilizing pathway [11]. Notably, mRNA levels of the foreign genes *cdt-1* and *gh1-1* from *N. crassa* seemed to increase in response to cellobiose (see Additional file 4: Figure S3), although these two genes were under the control of the *PGK1* promoter, and endogenous *PGK1* expression levels were nearly unchanged in the cellobiose and glucose conditions (see Additional file 4: Figure S3). The heterologous cellobiose-utilizing pathway genes *gh1-1* and *cdt-1* were expressed at moderate levels, which might contribute to slow fermentation rates on cellobiose. To test whether or not increased expression of *cdt-1* and/or *gh1-1* would improve cellobiose fermentation, mRNA quantification analysis by RNA deep sequencing (see Additional file 1: Dataset S1) was used to determine which genes are highly expressed on cellobiose. The three genes with the highest expression on cellobiose were *CCW12*, *TDH3*, and *FBA1* (Figure 4A; see Additional file 1: Dataset S1), suggesting that the promoters for these genes might be useful to improve the expression levels of *gh1-1* and *cdt-1*, and further improve cellobiose fermentation. The promoter strengths for *CCW12*, *TDH3*, and *FBA1* were verified by flow cytometry of cells expressing CDT-1 fused with an enhanced green fluorescent protein (eGFP) from the different promoters (Figure 4B; see Additional file 4: Figure S4A). Additionally, expression of codon-optimized *gh1-1* (hereafter named *gh1-1a*) resulted in about 2-fold enhanced β-glucosidase activity in cell extracts (see Additional file 4: Figure S4B). To test for the best cellobiose fermentation pathway, 16 combinations of plasmids were created using the promoters P_*CCW12*_, P_*TDH3*,_ and P_*FBA1*,_ as well as the original promoter P_*PGK1*,_ to express *cdt-1* and *gh1-1a* (Figure 4C). Comparisons of strains harboring these plasmids indicated that the combination of P_*TDH3*_ for *cdt-1* expression and P_*CCW12*_ for *gh1-1a* resulted in the best-performing cellobiose-utilizing pathway, with approximately two-fold higher fermentation rates compared with the pathway driven by *PGK1* promoters (Figure 4D; see Additional file 4: Figure S4C). Interestingly, the cellobiose-utilizing pathways segregated in performance based on the promoter driving *gh1-1a* (see Additional file 4: Figure S4D), suggesting that expression levels of *gh1-1a* may be limiting in cellobiose fermentation.

### Combined effect of TF mutants and the best-performing cellobiose-utilizing pathway on cellobiose fermentation

**Figure 5:**
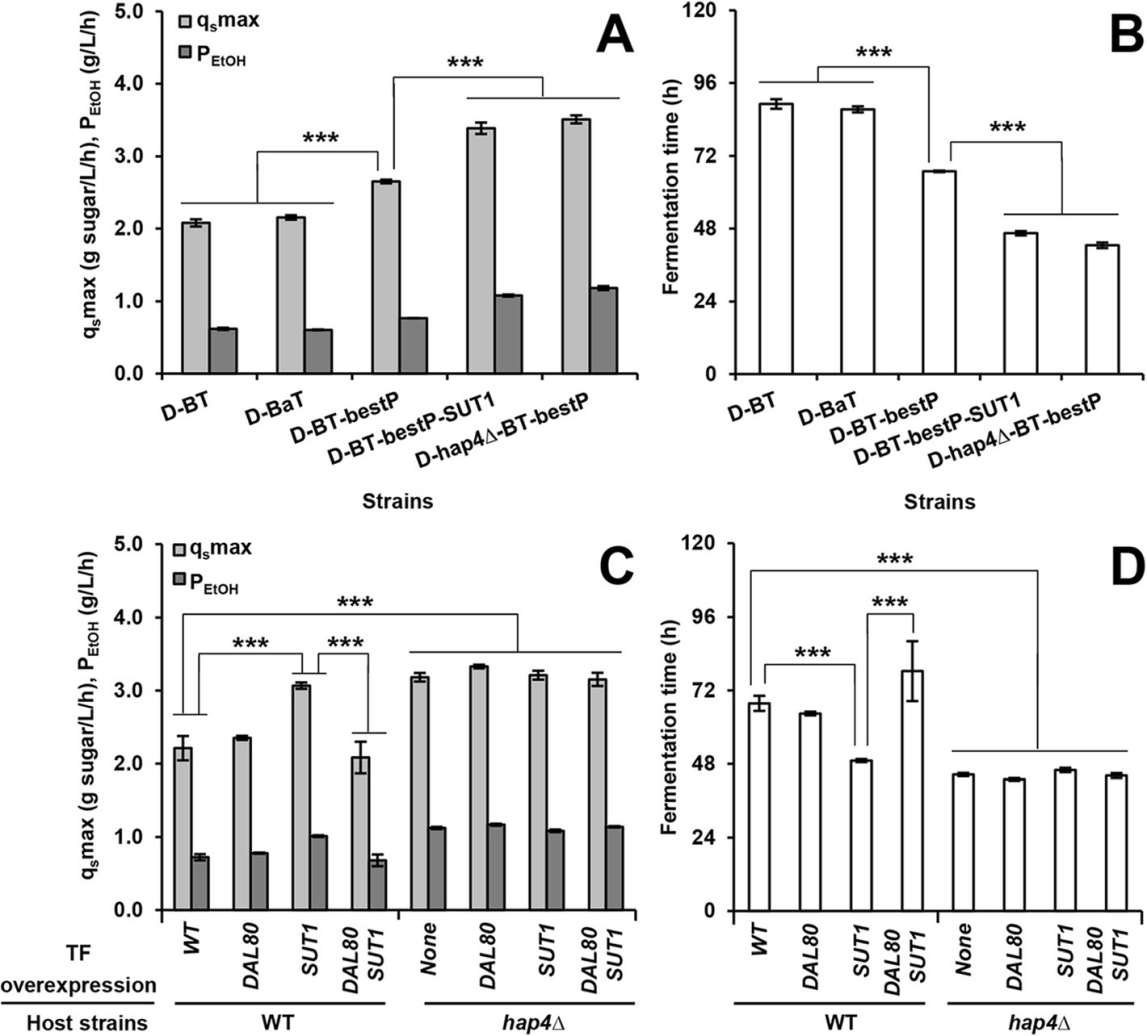
**Comparisons of cellobiose fermentation using strain D452-2 with the original (P**_***PGK1***_ **-driven** ***cdt-1*** **and** ***gh1-1a*** **) and optimized (P**_***TDH3***_ **-driven** ***cdt-1*** **and P**_***CCW12***_ **-driven** ***gh1-1a*** **) cellobiose-utilizing pathways and transcription factor (TF) mutants. (A)** Cellobiose-consumption rates and ethanol productivities with strains expressing the original and optimized cellobiose-utilization pathways, and also either overexpressing *SUT1* or harboring a *hap4* deletion. For details of the strains, see Additional file 4: Table S1. **(B)** Fermentation times of the strains in **(A)**. **(C)** Comparisons of cellobiose-consumption rates and ethanol productivities with WT D452-2 or D452-2 (*hap4Δ*) expressing the optimized cellobiose-utilization pathway, and additionally overexpressing *SUT1* and/or *DAL80*. **(D)** Fermentation times of the strains in **(C)**, defined as the time when ethanol reached its maximum titer. In all experiments in **(A–D)**, an initial OD_600_ of 20 was used. Data represent the mean and standard error of triplicate cultures on each source. Statistical analysis in **(A)** and **(C)** was performed using two-way ANOVA (with strains and fermentation rate including q_s_max and P_EtOH_ as the factors) followed by Tukey’s multiple-comparison posttest (***P < 0.001). Statistical analysis in **(B)** and **(D)** was performed using one-way ANOVA followed by Tukey’s multiple-comparison posttest (***P < 0.001).

To evaluate the combined effect of TF mutants and optimization of the heterologous cellobiose-utilizing pathway, cellobiose fermentation of strains with the original (P_*PGK1*_-driven *cdt-1* and *gh1-1* or *gh1-1a*) and optimized (P_*TDH3*_-driven *cdt-1* and P_*CCW12*_-driven *gh1-1a*) cellobiose-utilizing pathways, as well as with additional *SUT1* overexpression or *HAP4* deletion, were compared (Figure 5A, B). Cellobiose fermentation experiments were performed using high-cell density inocula to assess the upper limits of cellobiose fermentation performance by the engineered strains. Strains with the original or codon-optimized *gh1-1* showed similar fermentation performance. The best cellobiose-utilizing pathway fine-tuned by promoter engineering resulted in an increase of 28% and 24% in cellobiose-consumption rate and ethanol productivity. *SUT1* overexpression or *HAP4* deletion further increased the cellobiose-consumption rates and ethanol productivities by about 30% and 48%, respectively. Overall, optimization of the cellobiose-utilizing pathway and TF perturbations additively increased cellobiose-consumption rates and ethanol productivities by approximately 66% and 83% (Figure 5A), respectively, and shortened the fermentation times by approximately 45 hours compared with the WT strain expressing the original cellobiose-consumption pathway (Figure 5B).

To test whether the effects of the TFs on cellobiose fermentation are synergistic, D452-2 strains overexpressing *DAL80* or *SUT1,* or with *HAP4* deletion, were combined with the best-performing cellobiose-utilizing pathway (Figure 5C, D). When P_*TDH3*_-driven *cdt-1* and P_*CCW12*_-driven *gh1-1a* were combined with *SUT1* overexpression, the cellobiose-consumption rate and ethanol productivity increased by 39% and 40%, respectively (Figure 5C), and the mutant strain finished fermentation approximately 19 hours earlier than the WT strain expressing the optimized cellobiose-consumption pathway (Figure 5D). However, *DAL80* overexpression in D452-2 resulted in similar cellobiose fermentation rates to those of the WT strain expressing the optimized cellobiose-consumption pathway, and negatively influenced the effect of *SUT1* overexpression when both *DAL80* and *SUT1* were overexpressed (Figure 5C, D). When D452-2 (*hap4∆*) was used as a host strain, similar fermentation profiles were observed regardless of whether *SUT1* or *DAL80* were overexpressed, and the positive effects on cellobiose fermentation were similar to *SUT1* overexpression. All the mutants using D452-2 (*hap4∆*) as the host strain had average increases of 46% and 56% in cellobiose-consumption rates and ethanol productivities, respectively (Figure 5C), and finished fermentation approximately 23 hours earlier than the WT D452-2 expressing the best cellobiose-utilization pathway (Figure 5D).

## Discussion

Production of renewable fuels and chemicals from plant cell wall using *S. cerevisiae* will require substantial engineering to enable this yeast to function on an industrial scale. For example, *S. cerevisiae* must be adapted to consume abundant non-glucose sugars such as cellobiose and xylose. Evolutionary engineering has been widely adopted to improve *S. cerevisiae* strains [60,64,65], but these approaches generate strains that are difficult to further engineer, for example, by crossing. Systems biology methods could provide a framework for rational strain engineering, as they report on physiological responses on a genome-wide and organism-wide scale. However, identifying causative effects from systems-level data remains a difficult problem. Here we show that transcription profiling can be used to identify the regulatory mechanisms underlying suboptimal metabolism and swiftly narrow down the targets for engineering *S. cerevisiae* to rapidly ferment cellobiose, a prototypical non-glucose sugar released from the plant cell wall in industrial processes.

In this study, transcription profile comparisons between cellobiose-fermenting and glucose-fermenting cultures provided a genome-wide view of differential gene expression, and thus uncovered how the GRNs in yeast are perturbed by the novel non-glucose sugar cellobiose (Figure 1B). The cAMP-dependent PKA pathway, which controls the majority of glucose-induced changes, was activated to an extent under cellobiose conditions, as the overwhelming majority of genes (91.8%) displayed no significant change in transcriptional expression compared with yeast grown on glucose (see Additional file 1: Dataset S1, absolute fold changes ≥2.0, *P* ≤ 0.001). The decreased expression of *RGS2* and *PDE1* on cellobiose suggests that cAMP-mediated signaling in cellobiose-grown cells might be similar to that in glucose-grown cells (see Additional file 2: Dataset S2) [66,67]. In the presence of glucose, *HXT1*, *HXT3*, *STD1*, and *MIG2* were relieved from the repression complex of Rgt1 (see Additional file 4: Figure S5A) [68]. All of these genes were repressed in the presence of cellobiose, and *HXT6* and *HXT7*, which require Snf3 to be repressed by the Snf1-Mig1 glucose repression pathway, were de-repressed in the presence of cellobiose (see Additional file 2: Dataset S2; see Additional file 4: Figure S5A). This observation suggests that, in contrast to extracellular glucose, extracellular cellobiose is not sensed by Snf3 and Rgt2 to regulate glucose transport. Snf3 and Rgt2 were reported to sense extracellular xylose [69], suggesting that the GRNs involved in sugar transport have varied responses to different non-glucose sugars. Many genes involved in respiration, gluconeogenesis, and the metabolism of alternative carbon sources and their TFs are repressed by the Snf1-Mig1 glucose repression pathway, which allows aerobic fermentation and the preferential use of glucose when cells are grown in mixed sugars [17]. Strikingly, the expression of these genes increased in cellobiose-grown cells (see Additional file 2: Dataset S2, Additional file 4: Figure S5B). This result indicates that the Snf1-Mig1 glucose repression pathway is not activated by cellobiose as it is by glucose. A similar situation may also occur in recombinant xylose-utilizing *S. cerevisiae* [70], although additional aspects of xylose also play a role. For instance, Hxk2 has dual functions as a glycolytic enzyme and a regulator of the glucose repression pathway [71]. Xylose can inactivate Hxk2 through an autophosphorylation mechanism because it lacks C6 hydroxyl, which impedes all functions of Hxk2 [72,73]. A Hxk2 variant (Phe159Val) showed increased catalytic activity in the presence of xylose, which improved xylose fermentation by potentially restoring the regulatory function of Hxk2 [74]. By contrast, cellobiose is unlikely to inactivate Hxk2, which suggests that Hxk2 might not be a key factor responsible for inactivation of the glucose repression pathway in cellobiose conditions. Taken together, potential limitations for rapid cellobiose metabolism in terms of GRNs were observed in multiple glucose-responsive pathways (Figure 1; see Additional file 4: Figure S5). Future experiments will be required to determine how cellobiose perturbs the GRNs at the molecular level in engineered *S. cerevisiae*.

Using transcription profiling, we were able to identify pathways differentially regulated in cellobiose versus glucose fermentations, including mitochondrial function and amino acid biosynthesis. Interestingly, similar patterns of pathway activation and repression were seen with xylose [65]. However, how to apply these new insights to further improve the *S. cerevisiae* strains is not obvious. By using transcription profiling to identify TFs that are significantly differentially expressed in the two fermentation conditions, we were able to rapidly screen a small number of targets for improved cellobiose utilization. Further, transcription profiling provided quantitative data on transcription promoter strengths under the actual conditions of cellobiose fermentation. These data allowed us to rationally test defined promoter strengths for the heterologous cellobiose-consumption pathway. By combining TF engineering with promoter screening, we improved cellobiose fermentation rates by over 60% (Figure 5A) and fermentations finished around 45 hours earlier than those containing the original engineered yeast strain (Figure 5B), without affecting ethanol yield (see Additional file 4: Figure S6).

Our strategy also identified at least two candidate TFs whose expression levels might be generally engineered in *S. cerevisiae* strains to improve cellobiose fermentation. *SUT1*, which improved anaerobic cellobiose fermentation when overexpressed, is an activator of sterol uptake and hypoxic genes [75], but its regulatory role in central carbon metabolism is not known. Constitutive overexpression of *SUT1* has been shown to slow growth on glucose in aerobic cultures by downregulating genes important for respiratory metabolism [76], and it was also shown to block filamentous growth [77]. *HAP4*, deletion of which improved anaerobic cellobiose fermentation is a regulatory subunit of the Hap2/3/4/5 complex that positively regulates respiratory gene expression, and is a hotspot of genetic variation in cell physiology between yeast strains [27]. *HAP4* overexpression has been reported to positively affect the balance between respiration and fermentation of glucose metabolism in aerobic conditions by inducing mitochondrial function [78]. In contrast to *HAP4* deletion, which increased cellobiose fermentation, *HAP4* overexpression resulted in contradictory effects in different strain backgrounds. Interestingly, Sut1 and Hap4 are involved in a complex interplay between TFs including Cat8, Sip4, Adr1, and Rds2, which regulate the utilization of non-fermentable carbon sources [79]. *DAL80* overexpression shortened the lag phase of cellobiose fermentation (Figure 3D, E), but reduced the positive effect of *SUT1* overexpression (Figure 5). Dal80 is one of four regulators in NCR [47]. NCR connects to glucose signaling through the action of the NCR regulator Gln3 [80], but the role of Dal80 in the regulation of sugar metabolism is unknown. Although it is not entirely clear how the three TFs (Sut1, Hap4, and Dal80) regulate cellobiose fermentation, the pathways we identified, including those for mitochondrial function and amino acid metabolism, shed new light on how non-glucose sugars affect *S. cerevisiae* physiology, and could serve as a starting point for further engineering efforts. Even if these TFs prove to be beneficial only in a narrow host range [27,28], the systems-level strategy we employed should be useful in other *S. cerevisiae* strains to identify a narrow range of targets for strain improvement.

Although we focused on transcription profiling as a starting point for rational strain improvement, it is clear that multiple layers of post-transcriptional regulation, including that of TFs, probably also contribute to suboptimal non-glucose sugar fermentation. These additional layers of regulation could be exploited in the future to further improve non-glucose sugar fermentation. For example, central carbon metabolism is thought to be highly regulated at the post-transcriptional level [81]. Additional methods such as ribosome profiling [82] and metabolomics [83] could be used to identify additional bottlenecks in central carbon metabolism that could be addressed in the future. These methods to address post-transcriptional regulation could also be used to identify a small number of targets that could be engineered to improve co-fermentation of plant cell wall-derived sugars (such as cellobiose and xylose, and to improve the production of “drop-in” fuels and other renewable chemicals using yeast.

## Conclusions

Our systems-level study has revealed some of the key regulatory mechanisms underlying suboptimal metabolism of the non-glucose sugar cellobiose, and further optimized cellobiose fermentation by engineered *S. cerevisiae*. Compared with glucose metabolism, cellobiose metabolism induced mitochondrial activation and reduced amino acid biosynthesis under fermentation conditions. Using systems-level inputs as a starting point, we were able to modulate key TFs that cause suboptimal cellobiose fermentation and fine-tune the expression of the heterologous cellobiose-consumption pathway, greatly improving cellobiose fermentation by engineered *S. cerevisiae*. Thus, our study demonstrates a promising paradigm for systems biology-based rational microbial strain engineering.

## Methods

### Plasmids and *S. cerevisiae* stains

For all plasmids and host strains used in this study, see Additional file 4: Table S1. The RF-cloning method [84] and In-Fusion® HD Cloning Kit (Clontech Laboratories, Inc., Mountain View, CA, USA) were used for constructing plasmids. Genes for *cdt-1* and *gh1-1* from *N. crassa* [85] were used to reconstitute an intracellular cellobiose-utilization pathway in *S. cerevisiae*. eGFP-tagged *cdt-1* and *gh1-1* expressed from *PGK1* promoters were combined into a single plasmid using pRS426 (*2 μ URA*) [86] or pRS316 (*CEN URA*) [87] as backbone. The resulting plasmids were named pRS426-BT or pRS316-BT, respectively, and were transformed into *S. cerevisiae* strains to achieve intracellular utilization of cellobiose. The genes for TFs were inserted into the pRS313 (*CEN HIS*) [87] plasmid under the control of the *TEF1* promoter and *CYC1* terminator (for primers, see Additional file 4: Table S2). CEN plasmids were used to minimize variation due to variable plasmid copy number.

Strain BY4742 and TF knockout mutants (Open Biosystems Co., Lafayette, CO, USA), D452-2 [59], and a uracil auxotroph of the SR8 strain [60] (which is an engineered xylose-fermenting strain derived from D452-2) were used as host strains as specified in the text. Yeast transformations were performed (Frozen-EZ Yeast Transformation II Kit™; Zymo Research Corp., Irvine, CA, USA) in accordance with the instructions. To select transformants using an amino acid auxotrophic marker, the appropriate complete minimal dropout medium was used, which contains 43.7 g/l drop out base with agar (DOBA), double the recommended amount of the appropriate complete supplement mixture (CSM) dropout mixture (MP Biomedicals, Santa Ana, CA, USA) and 100 mg/l adenine hemisulfate.

To delete TFs in strain D452-2, a PCR-based gene disruption method was used [88]. The *KanMX* expression cassette was amplified by PCR (for primers, see Additional file 4: Table S2) that target the *KanMX* expression cassette on the plasmid pUG6 [88] and include 50 bp sequences homologous to upstream and downstream sequences of the *TF* gene. Positive transformants were selected on YPAD medium (10 g/l yeast extract, 20 g/l Bacto peptone, 100 mg/l adenine hemisulfate and 20 g/l of glucose) with 200 μg/ml of geneticin G418. Diagnostic PCR reactions with primers targeting approximately 200 bp upstream of the TF gene and the *KanMX*-specific primer (KanB), or specific primers (see Additional file 4: Table S2) targeting the middle of the *TF* gene, were used to confirm successful deletion.

### Anaerobic fermentation

Flask fermentations were performed under anaerobic conditions using optimal minimal medium (OMM), which was a modified version of previous defined media compositions [89,90], and contained (per liter) 1.7 g yeast nitrogen base (YNB) lacking ammonium sulfate (catalog number Y1251; Sigma-Aldrich, St. Louis, MO, USA), two-fold appropriate CSM dropout mixture (MP Biomedicals, Santa Ana, CA, USA), 10 g (NH4)_2_SO_4_, 1 g MgSO_4_ · 7H_2_O, 6 g KH_2_PO_4_, 100 mg adenine hemisulfate, 10 mg inositol, as well as an additional 100 mg glutamic acid, 20 mg lysine, and 375 mg serine. In addition, 80 g/l of glucose, 80 g/l cellobiose or 40 g/l xylose were added as carbon sources. The pH of each medium was adjusted to 6.0 and buffered using 100 mM 4-morpholineethanesulfonic acid (MES monohydrate; Sigma-Aldrich, St. Louis, MO, USA). When the initial OD of approximately 1.0 was used for setting up fermentation experiments, the medium was supplemented with 0.42 g/l Tween 80 and 0.01 g/l ergosterol.

Yeast cells transformed with plasmids expressing the cellobiose-utilization pathway and showing green fluorescent protein (GFP) fluorescence on plates were grown in 50 ml Falcon tubes containing 10 ml OMM media with 20 g/l glucose at 30°C for 24 hours. Cells were then inoculated to prepare starting cultures in 500 ml flasks. Cells were harvested in the early stationary phase and inoculated after washing with sterile double-distilled H_2_O. The initial OD_600_ of yeast used for anaerobic fermentations was around 1 or 20, as specified in the text. To achieve anaerobic conditions, cells were grown at 30°C with shaking at 220 rpm in 150 ml sealed serum flasks containing 50 ml media. The flasks were sealed with a rubber stopper clamped with an aluminum seal, then nitrogen gas purging was carried out for 20 minutes. Samples were taken through the stopper via a sterile syringe.

### RNA sequencing and data analysis

Anaerobic cultures of strain BY4742 expressing the cellobiose-utilizing pathway from plasmid pRS426-BT were carried out in biological triplicate using either 80 g/l glucose or 80 g/l cellobiose. Samples were harvested when approximately 50 g/l of residual sugar was present (Figure 1A). Cells were harvested in Falcon tubes precooled in liquid N_2_ by centrifuging for 5 minutes at 4000 rpm (3220 × g). The supernatant was discarded, and the pellet was stored at −80°C until further use.

Total RNA was extracted using the RiboPure Yeast kit (Ambion, Austin, TX, USA), in accordance with the manufacturer’s instructions, except that cells were disrupted by bead-beating three times for 30 seconds each, with a pause for 30 seconds pbetween runs. Total RNA (4 μg) was used to prepare the multiplexing libraries with barcodes (TruSeq RNA Sample Prep Kit; Illumina) following the manufacturer’s instructions. The final cDNA libraries were quantified (Agilent Bioanalyzer 2000; Functional Genomics Laboratory, University of California, Berkeley, CA, USA) and sequenced (Illumina Genome Analyzer-II ; Vincent J. Coates Genomic Sequencing Laboratory, University of California, Berkeley) using standard Illumina operating procedures.

Sequence reads were assembled and analyzed in CLC Genomics Workbench 6.5 (CLC Bio, Aarhus, Denmark). The *S. cerevisiae* S288C genome was downloaded from RefSeq at the NCBI (sequence assembly version R64-1-1) [91] including 16 chromosomes and the mitochondrial genome. The genes for *N. crassa* β-glucosidase *gh1-1* and eGFP-tagged *N. crassa* cellodextrin transporter *cdt-1* as encoded in pRS426-BT were manually annotated and combined with the *S. cerevisiae* S288C genome as the reference (total size of 12.17 Mb). Expression values were normalized by calculating the reads per kb of mRNA exon per million mapped reads (reads per kb per million; RPKM), and further normalized using the option of “By totals” [92]. A mean of 38 million 50 bp single reads corresponding to approximately 156-fold coverage of the reference was generated for each library. Following the default parameters in the CLC Genomics Workbench, around 92.3% of reads per library was successfully imported, of which approximately 83.4% was mapped. Of the imported reads, about 76.3% was uniquely mapped. Next, an unpaired two-group comparison of all six libraries using the mapping results was used for quality control analysis. The tools for quality control including box plots and hierarchical clustering showed that the biological triplicate libraries were grouped according to the different sugar conditions (see Additional file 4: Figure S7A-B). Transcription profile data are available in supplementary material (see Additional file 1: Dataset S1) and from the Gene Expression Omnibus (accession no.GSE54825) [93]. All annotations were derived from the SGD gene association file [94].

To identify differential expression in the cellobiose versus glucose fermentations, an unpaired two-group comparison was used to generate fold changes and further analyzed for statistical significance using Baggerley’s test [95]. Significantly differentially expressed genes, highlighted in red in volcano plots (see Additional file 4: Figure S7C), were then sorted by applying a stringent false discovery rate (FDR)-corrected cutoff *P*-value of 0.001 or less [96] and an absolute fold-change threshold of 2.0 or greater. Significantly upregulated and downregulated genes (see Additional file 2: Dataset S2) were further tested for GO biological process enrichment using FunSpec with a *P*-value cutoff of 0.01 and Bonferroni correction [97,98] (see Additional file 3: Dataset S3).

### Optimization of the cellobiose-utilizing pathway using codon-optimized *GH1-1* and promoter engineering

To improve its expression in *S. cerevisiae*, *gh1-1* was codon-optimized by DNA2.0 (USA), and is hereafter named *gh1-1a* (see Additional file 4: Figure S8). The three genes with the highest expression on cellobiose (*CCW12*, *TDH3*, and *FBA1*), as determined from the RNA deep sequencing data, were identified and further confirmed for promoter strength by overexpressing the eGFP-tagged CDT-1 (see Additional file 1: Dataset S1). A mutant pathway library was created by combining the two cellobiose-utilizing genes *cdt-1* and *gh1-1a* and four promoters (P_*CCW12*_, P_*TDH3*_, P_*FBA1*_ and P_*PGK1*_), and screened in strain D452-2 for cellobiose fermentation (Figure 4C). The eGFP fused to CDT-1 was detected by excitation at 488 nm to confirm the promoter strengths. Cells from the mutant pathway library were harvested at mid-exponential phase, washed, and resuspended in 1 × phosphate buffered saline (PBS) pH 7.4. WT D452-2 was used as a negative control. The cells were analyzed and sorted (Cell Lab QUANTA* Flow Cytometer; Beckman Coulter, Brea, CA). Quantitative fluorescence surface density (FSD) was estimated from the raw data of EV (electronic volume) and FL1 (fluorescence, green) values (FSD = FL1/ ((volume channel) ^ (2/3))) [99].

### Analytical methods and calculation of fermentation parameters

Cell growth was monitored at OD_600_ using a UV-visible spectrophotometer (8453 UV–vis; Agilent, Santa Clara, CA, USA). Cellobiose, glucose, xylose, and ethanol concentrations were determined by high performance liquid chromatography on a chromatograph (Shimadzu) equipped with a refractive index detector and a fast acid column 100 mm length × 7.8 mm internal diameter (RFQ; Phenomenex Inc., Torrance, CA, USA). The column was eluted with 0.01 N of H_2_SO_4_ at a flow rate of 1.0 ml/min at 55°C.

Fermentation parameters including cellobiose-consumption rate (q_s_max) and ethanol productivity (P_*EtOH*_) were calculated for the fermentation profiles using Origin 8 (Originlab®) [100,101]. The data for cellobiose-consumption and ethanol production were plotted using Origin 8 (Originlab®), and curves were fitted to a Boltzmann function, which is used to fit a curve with a sigmoidal shape. The equation used was:

$$ y=\frac{A_1-{A}_2}{1+{e}^{\left(x-{x}_0\right)/ dx}}+{A}_2 $$

where *A*_1_ is the initial value, *A*_2_ is the final value, *x*_0_ is the inflection point of the sigmoidal curve, and *dx* is the time constant. The slope at the inflection point (*x*_0_) indicates the maximum rate of fermentation. The value for the slope is:

$$ \frac{\left({A}_2-{A}_1\right)}{4 dx} $$

Thus, the equation used for calculating cellobiose-consumption rate (g cellobiose/l/h) is:

$$ {q}_s \max =-\frac{\left({A}_2-{A}_1\right)}{4 dx} $$

and the equation used for calculating ethanol productivity (g ethanol/l/h) is:

$$ {P}_{EtOH}=\frac{\left({A}_2-{A}_1\right)}{4 dx} $$

To calculate the lag times for cellobiose consumption and ethanol production, the different values at 12 hour intervals were obtained according to the fitting equation, then log_10_ transformed for plotting. An intercept between the linear curve and the time axis before *x*_0_ was defined as the lag time. An intercept between the linear curve and the time axis after *x*_0_ was defined as the fermentation time, at which point cellobiose has been depleted and ethanol reaches maximum titer. The mean rates or oag times for the WT strain in different batches of fermentation were used to normalize those of the TF mutant strains.

### Statistical significance tests

For comparison of fermentation between the original recombinant cellobiose-utilizing strain and further engineered strains, ANOVA was used, followed by Tukey’s multiple-comparison posttest with a 95% confidence interval. Statistics were performed by using SigmaPlot (version 11.0). The differences were considered significant at *P* < 0.001 [88].

## Additional files

Additional file 1:
**Dataset 1.** The transcription profiles of cellobiose-grown and glucose-grown cells of engineered *Saccharomyces cerevisiae*. Additional file 2:
**Dataset 2.** Significant differential expression of 519 genes, 19 transcription factor genes and 7 regulatory genes in GRNs in cellobiose-grown cells compared with glucose-grown cells of engineered *Saccharomyces cerevisiae* (absolute fold changes ≥2.0, *P* ≤ 0.001). Additional file 3:
**Dataset 3.** Gene Ontology Biological processes for significantly upregulated and downregulated genes. Additional file 4:
**Supporting information. **Figures S1-S8, Table S1, and Table S2.

## Abbreviations

CSM: Complete supplement mixture
DOBA: drop out base with agar
eGFP: enhanced green fluorescent protein
FDR: false discovery rate
FSD: fluorescence surface density, GFP, green fluorescent protein
GO: Gene Ontology
GRN: Gene regulatory network
NCR: nitrogen catabolite repression
OMM: Optimal minimal medium
PKA: Protein kinase A
TF: Transcription factor
WT: wild type
