# Supplementary material for: Leveraging transcription factors to speed cellobiose fermentation by Saccharomyces cerevisiae
Source: Biotechnol Biofuels. 2014 Aug 27;7:126. doi: 10.1186/s13068-014-0126-6 (PMC4243952; doi:10.1186/s13068-014-0126-6)
Supplement: Additional file 4: — Supporting information. Figures S1-S8, Table S1, and Table S2. [file 13068_2014_126_MOESM4_ESM.pdf]

Supporting Information

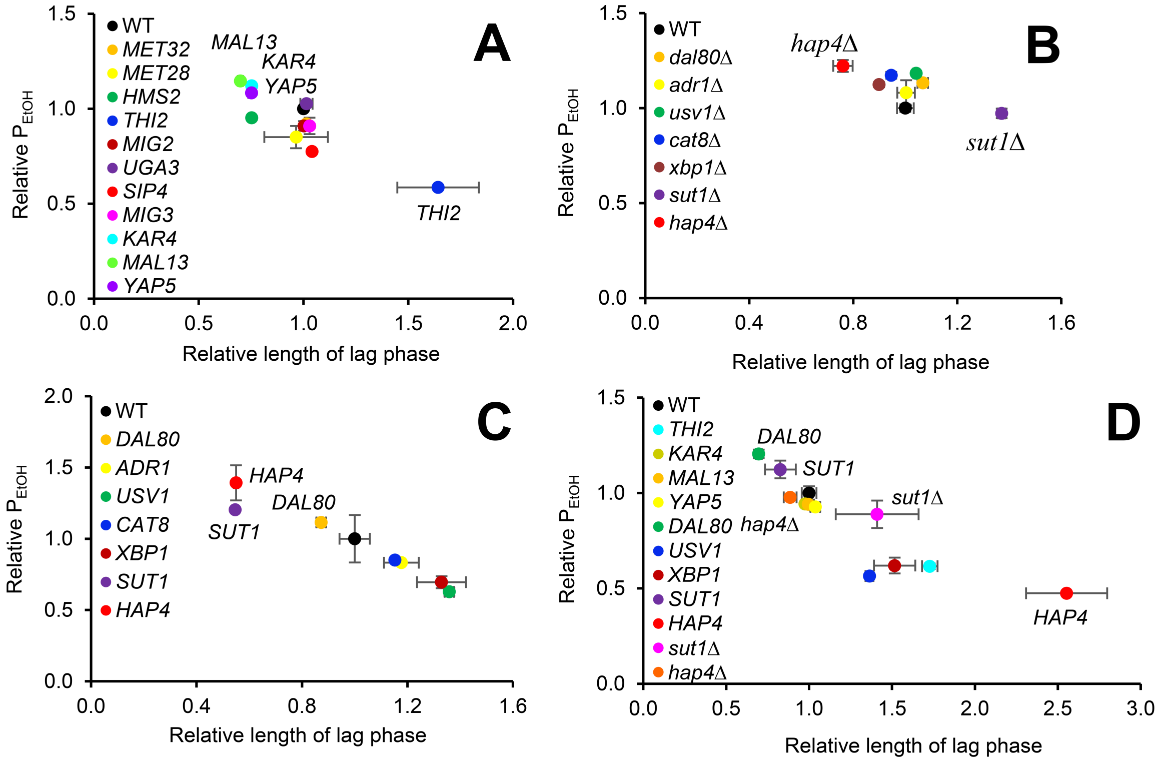

Figure S1. The effects of manipulating TFs on cellobiose fermentation.

Cellobiose fermentation by BY4742 with TF mutations and expressing the cellobiose utilization pathway from pRS316-BT. **(A)** Ethanol productivity using overexpression mutants of transcription factors with decreased expression levels on cellobiose, **(B)** deletion mutants or **(C)** overexpression mutants of transcription factors with increased expression levels on cellobiose. **(D)** Cellobiose fermentation using strain D452-2 with TF mutations and expressing the cellobiose utilization pathway from pRS316-BT. Relative ethanol productivities (P_{EtOH}) and the relative length of the lag phase were obtained by comparisons to the wild-type strain (normalized to 1.0). Plots of relative P_{EtOH} versus relative length of the lag time are shown. Each point represents duplicate anaerobic fermentations using a starting cellobiose concentration of 80 g/L and starting OD_{600} = 1. The ethanol productivity and length of the lag phase for the WT BY4742 strain were 0.41 (± 0.03) g/L/hr and 88.39 (± 4.72) hr, respectively, and for the WT D452-2 strain were 0.38 (± 0.01) g/L/hr and 76.23 (± 2.83) hr.

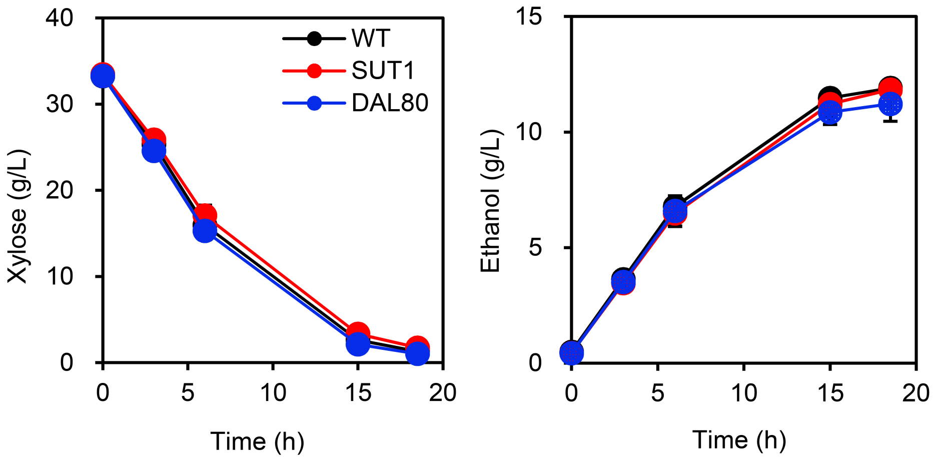

Figure S2. Fermentation profiles using SR8 (*ura3*Δ) with overexpressed SUT1 (red line) and DAL80 (blue line) compared with the wild type (black line).

(A) Xylose consumption; **(B)** Ethanol production.

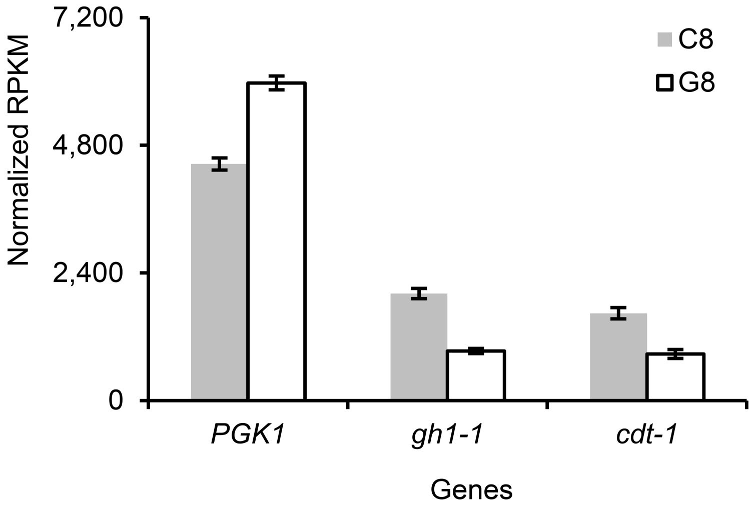

Figure S3. Transcriptional expression levels of the heterologous cellobiose-utilizing pathway in cellobiose-grown cells (C8) and glucose-grown cells (G8).

The p-values for difference in expression levels are: 1×10^{-17} for *PGK1*, 0 for *gh1-1*, and 1×10^{-7} for *cdt-1*.

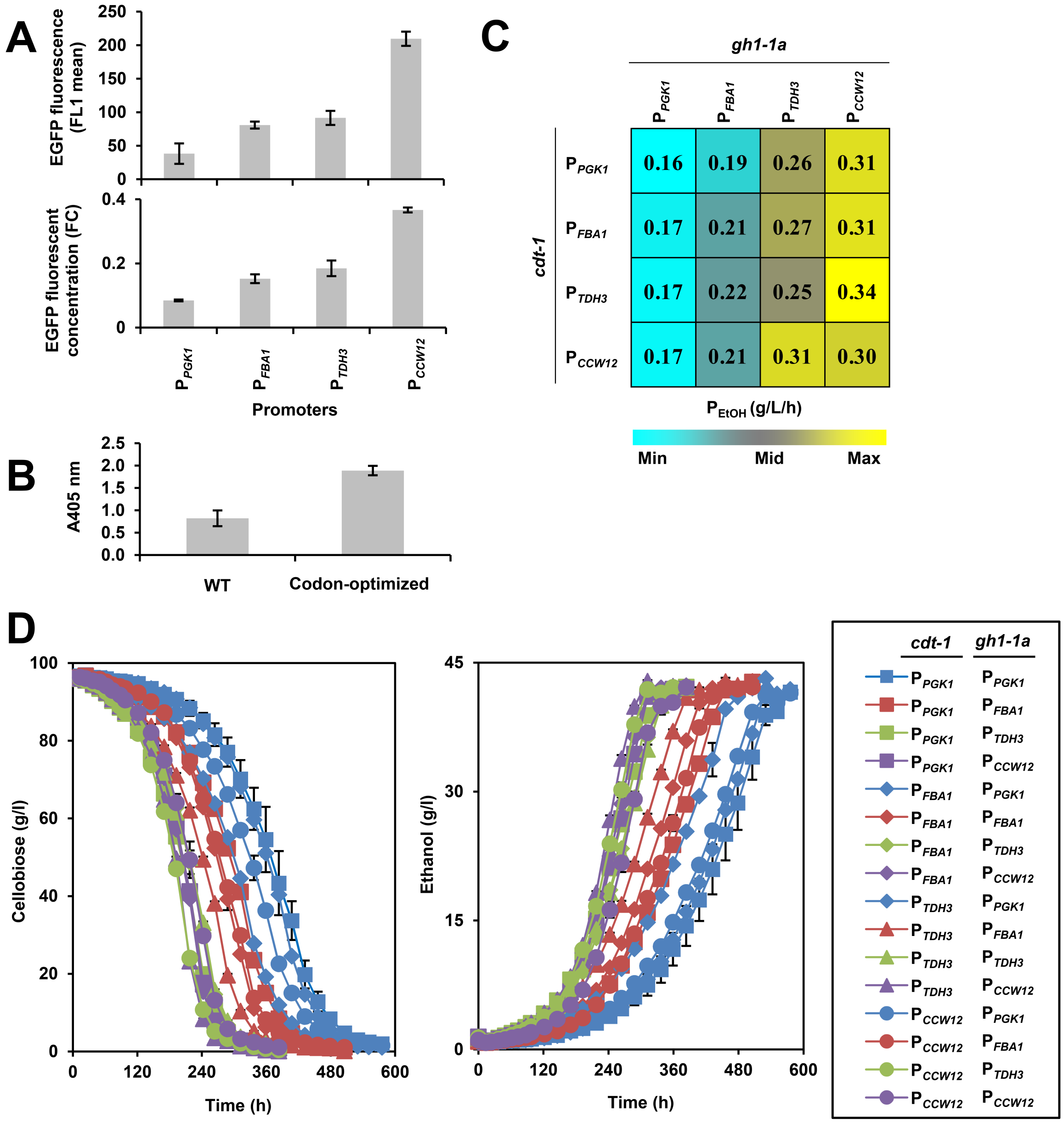

Figure S4. Optimization of *cdt-1* and *gh1-1* expression for cellobiose fermentation in *S. cerevisiae* from a promoter library.

(A) GFP fluorescence of *cdt-1* expressed from the different promoters in the promoter library; **(B)** β-glucosidase activities in 5 μg of cell extract with wild-type and codon-optimized GH1-1 (encoded by *gh1-1a*); **(C)** Comparison of ethanol productivity of *cdt-1* and *gh1-1a* expressed from promoters in the promoter library. **(D)** Fermentation profiles of cellobiose consumption (left panel) and ethanol production (right panel) of strain D452-2 expressing the cellobiose utilization pathway using the promoter library. A starting culture OD_{600} of 1 was used. Promoter combinations are shown to the right.

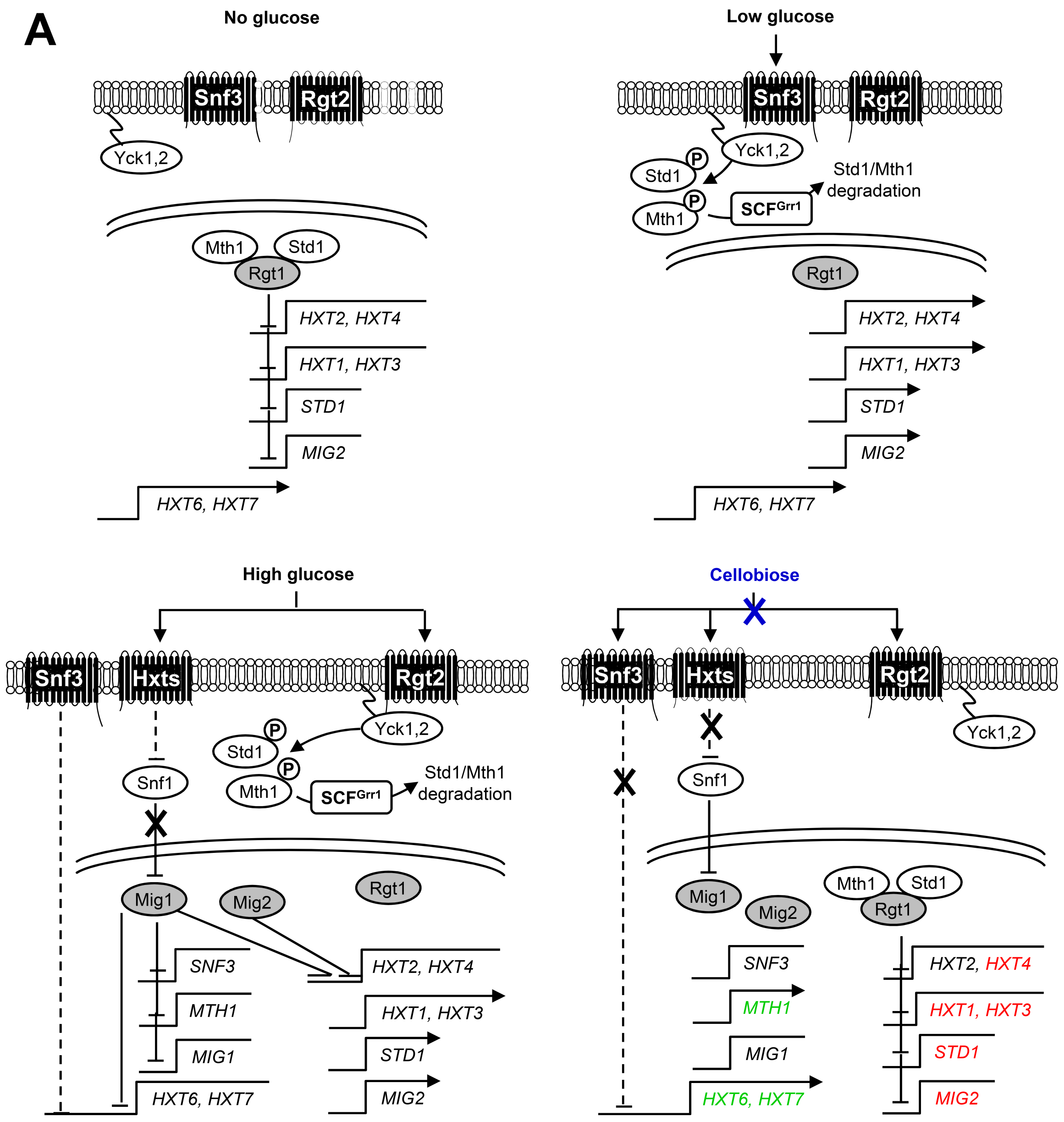
A
No glucose
Snf3
Rgt2
Yck1,2
Mth1
Std1
Rgt1
HXT2, HXT4
HXT1, HXT3
STD1
MIG2
HXT6, HXT7
Low glucose
Snf3
Rgt2
Yck1,2
P
Std1
P
Mth1
SCF^Grr1
Std1/Mth1
degradation
Rgt1
HXT2, HXT4
HXT1, HXT3
STD1
MIG2
HXT6, HXT7
High glucose
Snf3
Hxts
Rgt2
Yck1,2
P
Std1
P
Mth1
SCF^Grr1
Std1/Mth1
degradation
Snf1
Mig1
Mig2
Rgt1
SNF3
MTH1
MIG1
HXT6, HXT7
HXT2, HXT4
HXT1, HXT3
STD1
MIG2
Cellobiose
Snf3
Hxts
Rgt2
Yck1,2
Snf1
Mig1
Mig2
Mth1
Std1
Rgt1
SNF3
MTH1
MIG1
HXT6, HXT7
HXT2, HXT4
HXT1, HXT3
STD1
MIG2

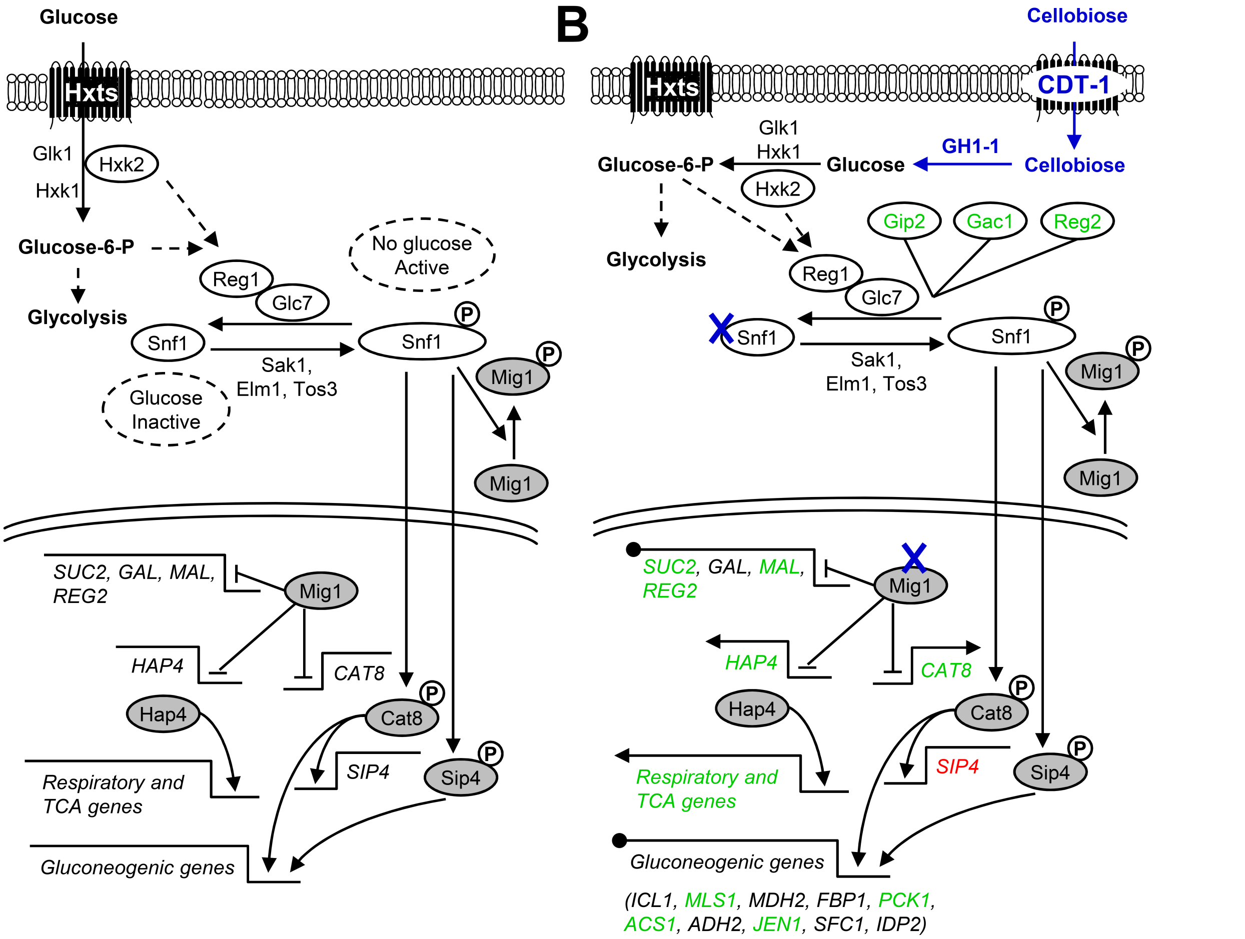

Figure S5. Proposed model for regulation mechanisms of cellobiose utilization by engineered *S. cerevisiae*.

(**A**) Misregulation of hexose transporters in cellobiose-grown cells. Regulation of *HXT* transporter gene expression in response to glucose (15, 16) and cellobiose are shown. (**B**) Partially inactive glucose repression pathway. The Snf1-Mig1 glucose repression pathway and gene expression of its targets are compared between in the presence of glucose (15) and cellobiose. In the presence of glucose, Snf1 is inactivated by mechanisms involving the Glc7-Reg1 complex that remain unclear (17, 18). The other regulatory subunits of Glc7–Gip2 (19) and Gac1 (19) that target Glc7 to various substrates for glycogen accumulation, and Reg2 (20) that targets Glc7 to substrates that are phosphorylated by Snf1 during glucose repression showed increased mRNA levels in cellobiose metabolism (Dataset S2). In both panels, down-regulated genes are highlighted in red, and up-regulated genes are highlighted in green.

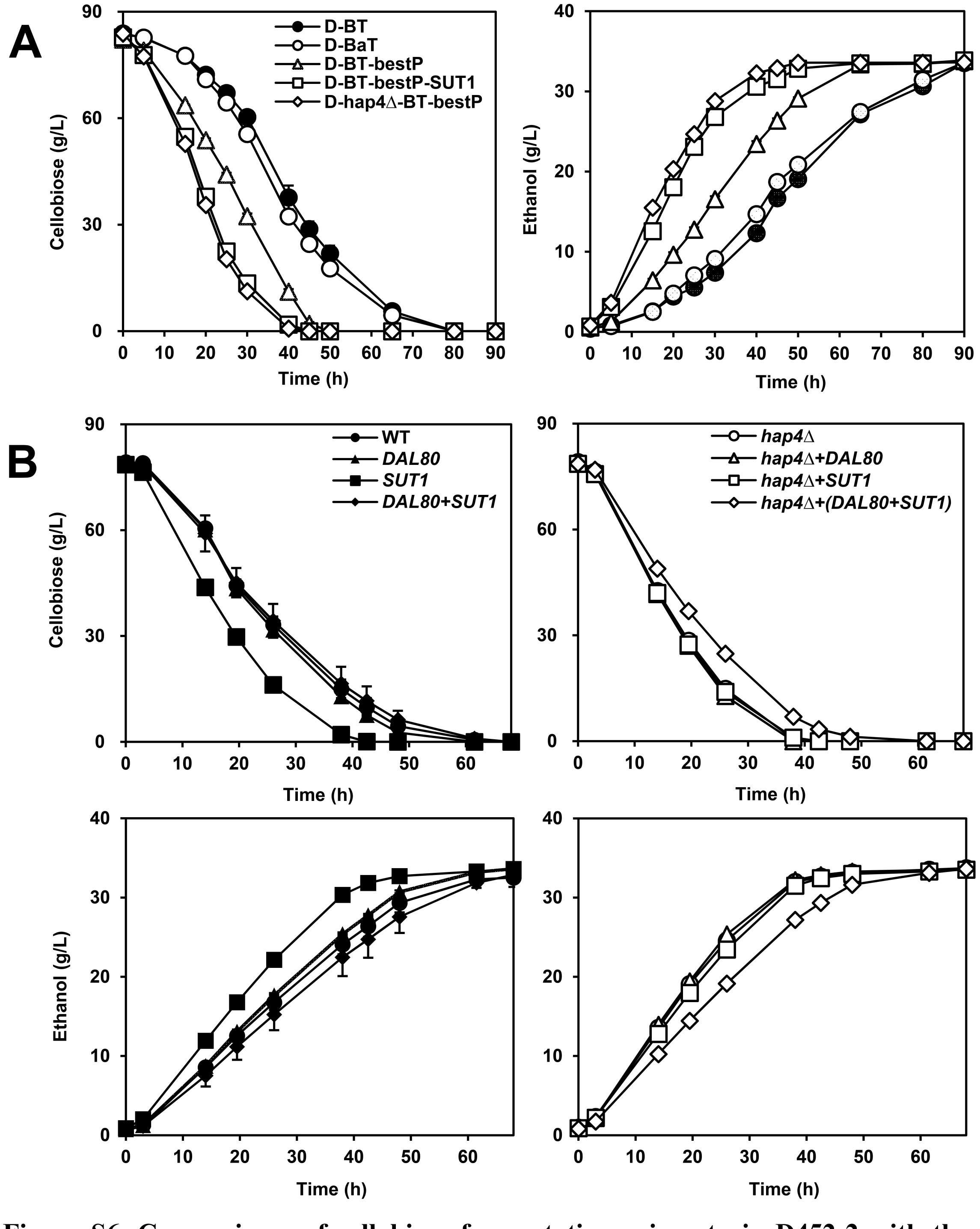

Figure S6. Comparisons of cellobiose fermentation using strain D452-2 with the original and optimized cellobiose-utilizing pathways and TF mutants.

(A) Cellobiose fermentation profiles with strains expressing the original and optimized cellobiose-utilization pathways, and additionally overexpressing *SUT1* or with a deletion of *hap4*. Strain information is listed in Table S1. **(B)** Cellobiose fermentation profiles using D452-2

with an optimized cellobiose-utilizing pathway (P_{TDH3}-driven *cdt-1* and P_{CCW12}-driven *gh1-1a*) with different TFs, starting with initial $OD_{600} = 20$.

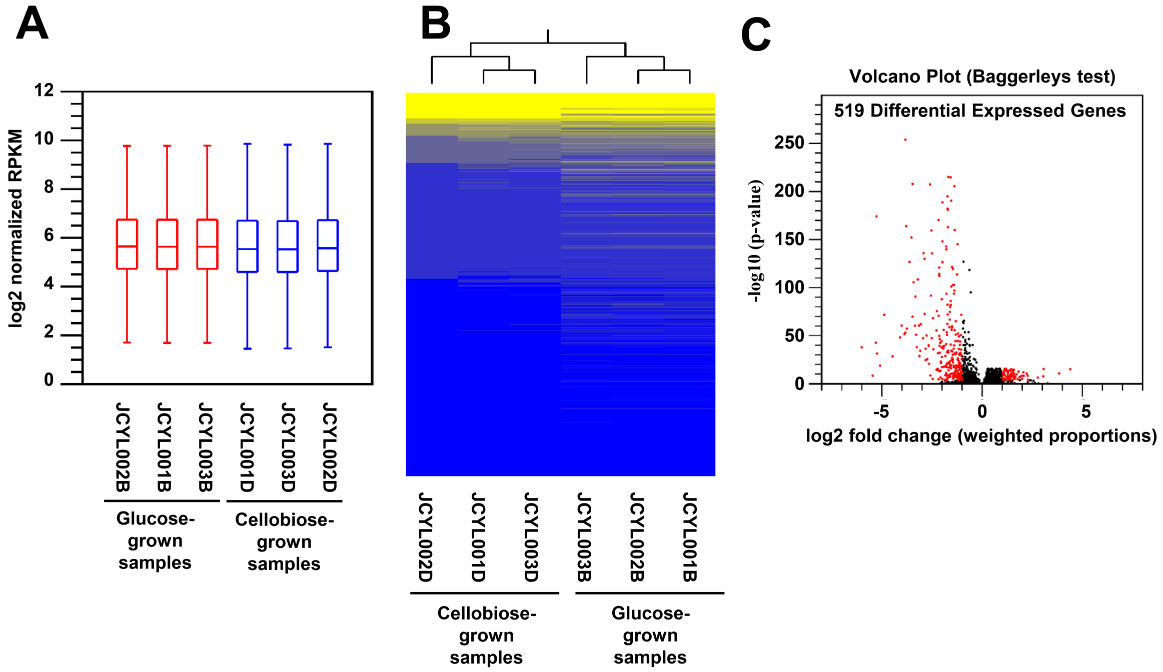

Figure S7 RNA-seq and expression analysis using CLC Genomics workbench.

(A) Box plot of 6 samples in color-coded 2 groups; **(B)** Hierarchical clustering of 6 samples with 2 clusters; **(C)** Statistical analysis to identify significantly differential expressed genes colored in read in volcano plots. The criteria were (FDR)-corrected cut off p value ≤ 0.001 and absolute fold changes threshold ≥ 2.0, correspondingly -log (p value) ≥ 3 and absolute $\log_2$ fold changes ≥ 1.0.

ATGTCTTTGCCAAAAGATTTTCTATGGGGTTTCGCTACCGCAGCCTATCAAATTGAGGGCGCTATTCACG
CTGATGGCAGAGGACCATCAATTTGGGATACATTTTGCAATATCCCAGGTAAGATAGCTGACGGATCTTC
CGGAGCTGTGGCATGTGACTCATACAACAGAACAAAAGAGGATATCGACTTATTGAAATCACTTGGGGCA
ACTGCTTACAGATTCTCTATTAGTTGGTCTAGAATCATTCCAGTAGGGGGCAGAAATGACCCTATCAATC
AGAAGGGTATTGATCATTACGTTAAGTTTGTGGATGATCTTTTGGAAGCTGGGATAACTCCATTCATCAC
ATTGTTCCATTGGGATTTGCCAGATGGTTTGGACAAAAGATACGGAGGCTTACTAAACAGGGAAGAGTTT
CCACTAGATTTTGAACACTACGCTAGAACCATGTTCAAAGCTATCCCTAAGTGTAAACATTGGATTACCT
TTAACGAACCTTGGTGTTCCTCAATACTTGGTTACAATTCTGGTTACTTTGCACCAGGGCATACATCTGA
CCGTACTAAGAGTCCAGTTGGCGATTCAGCTAGAGAACCATGGATTGTAGGCCATAACTTACTTATCGCA
CACGGAAGAGCTGTTAAAGTGTATAGAGAAGATTTCAAGCCTACTCAAGGTGGCGAGATCGGAATAACTC
TGAATGGTGACGCCACACTGCCATGGGACCCAGAAGATCCATTAGACGTTGAAGCCTGTGATCGTAAGAT
AGAATTTGCCATATCATGGTTTGCAGATCCTATCTACTTCGGAAAGTACCCAGATTCTATGAGAAAACAG
TTAGGTGACAGACTACCAGAGTTTACTCCTGAGGAGGTTGCTTTGGTGAAAGGTAGTAATGACTTCTATG
GTATGAACCATTACACAGCAAACTACATCAAACACAAAAAGGGGGTTCCACCAGAAGATGACTTCCTGGG
TAATCTGGAAACCTTATTCTATAACAAAAAGGGAAACTGCATCGGTCCTGAAACACAATCCTTCTGGTTA
AGACCACACGCCCAAGGTTTTAGAGATCTTTTGAATTGGTTGTCTAAGCGTTACGGATACCCTAAGATCT
ACGTTACAGAAAACGGTACATCTTTGAAAGGCGAAAATGCAATGCCTTTGAAACAAATTGTTGAGGATGA
CTTTAGGGTAAAGTACTTCAATGATTACGTCAATGCCATGGCTAAAGCTCATTCCGAAGATGGCGTGAAT
GTCAAAGGGTACTTAGCATGGAGTTTGATGGATAACTTTGAGTGGGCCGAAGGATATGAAACAAGATTTG
GCGTCACTTACGTAGATTATGAAAATGACCAAAAGAGATACCCAAAGAAATCAGCCAAATCCCTTAAACC
TCTATTCGATTCACTAATCAAAAAGGAC**CATCATCATCATCATCAT**TAA

Figure S8. The DNA sequence of codon-optimized *gh1-1* (*gh1-1a*) with a C-terminal His_6 tag. The sequence encoding the His_6 tag is in bold.

Table S1. Plasmids and *S. cerevisiae* strains used in this study

Plasmid or strain	Description	Reference or source
Plasmids		
pRS426	*URA3*, 2-μm origin	(3)
pRS426-BT	*N. crassa gh1-1* and *cdt-1* under the control of *PGK1* promoter and *CYC1* terminator in pRS426	This study
pRS316	*URA3*, ARS/CEN origin	(4)
pRS316-BT	*N. crassa gh1-1* and *cdt-1* under the control of *PGK1* promoter and *CYC1* terminator in pRS316	This study
pRS316-BaT	Codon-optimized *gh1-1a* and *cdt-1* under the control of *PGK1* promoter and *CYC1* terminator in pRS316	This study
pRS316-BT-best	The best-tuned cellobiose-utilizing pathway in pRS316 composed of P_{TDH3}-driven *gh1-1a* and P_{CCW12}-driven *cdt-1*	This study
pRS313	*HIS3*, ARS/CEN origin	(4)
pRS313-TEF1-CYC1	*HIS3*, CEN plasmid, *TEF1* promoter and *CYC1* terminator, used for making the constructs to overexpress TFs	This study
pRS315	*LEU2*, ARS/CEN origin, used for overexpressing codon-optimized *gh1-1a*	(4)
S. cerevisiae strains		
BY4742	MATalpha his3Δ1 leu2Δ0 lys2Δ0 ura3Δ0	Open Biosystems
D452-2	MATalpha, *leu2*, *his3*, *ura3*, and *can1*	(5)
D452-2 (*hap4*Δ)	MATalpha, *leu2*, *his3*, *ura3*, and *can1*, *hap4*::KanMX	This study
D452-2 (*sut1*Δ)	MATalpha, *leu2*, *his3*, *ura3*, and *can1*, *sut1*::KanMX	This study
SR8	ald6Δ of the evolved strain D452-2 *leu2::LEU2* P_{TDH3}-*XYL1*-T_{TDH3}; *ura3::URA3* P_{TDH3}-*XYL1*-T_{TDH3} P_{PGK1}-*XYL2*-T_{PGK1} P_{TDH3}-*XYL3*-T_{TDH3}; *his1::HIS1* P_{PGK1}-*XYL2*-T_{PGK1} P_{TDH3}-*XYL3*-T_{TDH3}	(6)
SR8 (*ura3*Δ)	Evolved xylose-utilizing strain derived from D452-2, auxotrophic for uracil	(6)
D-BT	D452-2 expressing *gh1-1* and *cdt-1* under the control of *PGK1* promoter in pRS316-BT and empty vector pRS313-TEF1-CYC1	This study
D-BaT	D452-2 expressing codon-optimized *gh1-1a* and *cdt-1* under the control of *PGK1* promoter in pRS316-BaT and empty vector pRS313-TEF1-CYC1	This study
D-BT-bestP	D452-2 expressing the best-tuned cellobiose-utilizing pathway in pRS316-BT-best composed of P_{TDH3}-driven *gh1-1a* and P_{CCW12}-driven *cdt-1* and empty vector pRS313-TEF1-CYC1	This study
D-BT-bestP-SUT1	D452-2 expressing the best-tuned cellobiose-utilizing pathway in pRS316-BT-best and overexpressed *SUT1* in pRS313	This study
D-*hap4*Δ-BT-bestP	D452-2 *hap4* deletion strain expressing the best-tuned cellobiose-utilizing pathway in pRS316-BT-best	This study

Table S2. Primers used in this study

Name	Sequence (5' → 3')	Application
Primers for cloning into pRS313 vector		
pRS313-F-2	ACTAGTTCTAGAAAACTTAGATTAGATTGCTATGCTTTCTTTCTAATG	PCR amplification of linear pRS313-TEF1-CYC1 from SpeI to XhoI
pRS313-R-2	CTCGAGTCATGTAATTAGTTATGTCACGCTTACATTCAC	
MET32-313 SpeI F	TTTTCTAGAACTAGTATGGAGGATCAGGATGCTGC	MET32 overexpression
MET32-313 XhoI R	ATTACATGACTCGAGTCAGCCATTACTGCTACCATTG	MET32 overexpression
MET28-313 SpeI F	TTTTCTAGAACTAGTATGAGTGCGAAACAAGGGTGG	MET28 overexpression
MET28-313 XhoI R	ATTACATGACTCGAGCTACCTGCCCATGTTCCGTCTC	MET28 overexpression
THI2-313 SpeI F	TTTTCTAGAACTAGTATGGTCAATAGTAAGAGGCAGCAGAG	THI2 overexpression
THI2-313 XhoI R	ATTACATGACTCGAGCTAGTCCTGCATGGCATATACATCC	THI2 overexpression
MIG2-313 SpeI F	TTTTCTAGAACTAGTATGCCTAAAAAGCAAACGAATTTCC	MIG2 overexpression
MIG2-313 XhoI R	ATTACATGACTCGAGTTAAACTCTTTTGGGACCGTTGAAAAC	MIG2 overexpression
UGA3-313 SpeI F	TTTTCTAGAACTAGTATGAATTATGGCGTGGAGAAGC	UGA3 overexpression
UGA3-313 XhoI R	ATTACATGACTCGAGTCAGGCAAAATTAATATTGTAATCTAAT	UGA3 overexpression
SIP4-313 SpeI F	TTTTCTAGAACTAGTATGGCCAAGAGGAAATATGGCAG	SIP4 overexpression
SIP4-313 XhoI R	ATTACATGACTCGAGTTAGAAGGTCGAGTTCAAAATATTCTGG	SIP4 overexpression
MIG3-313 SpeI F	TTTTCTAGAACTAGTATGAATTACCTGCGAGATAGATTTC	MIG3 overexpression
MIG3-313 XhoI R	ATTACATGACTCGAGTTATTTACTGAAGTAAGAAGGAGCACTG	MIG3 overexpression
HMS2-313 SpeI F	TTTTCTAGAACTAGTATGGATGCAACATCGAGGATGG	HMS2 overexpression
HSM2-313 XhoI R	ATTACATGACTCGAGTCACGTTCGAAGATGTTTGGAAAG	HMS2 overexpression
KAR4-313 SpeI F	TTTTCTAGAACTAGTATGGCATTCCAAGATCCAACTTACG	KAR4 overexpression
KAR4-313 XhoI R	ATTACATGACTCGAGTTATTGTGCTTTTTGTACTGGACTTCTTG	KAR4 overexpression
MAL13-313 SpeI F	TTTTCTAGAACTAGTATGACTTTAACTAAGCAAACATGCGC	MAL13 overexpression
MAL13-313 XhoI R	ATTACATGACTCGAGTCAAGGGTCTATGTCTTCATTATCCTTG	MAL13 overexpression
YAP5-313 SpeI F	TTTTCTAGAACTAGTATGGCTCTACCTCTGATAAAACCTAAGG	YAP5 overexpression
YAP5-313 XhoI R	ATTACATGACTCGAGTCAGTGGATGATGGACCGGAT	YAP5 overexpression
DAL80-313 SpeI F	TTTTCTAGAACTAGTATGGTGCTTAGTGATTCGTTGAAGC	DAL80 overexpression
DAL80-313 XhoI R	ATTACATGACTCGAGTTAATGTTGTGGGTGAGATTGTACTGAAG	DAL80 overexpression
ADR1-313 SpeI F	TTTTCTAGAACTAGTATGGCTAACGTAGAAAAACCAAACG	ADR1 overexpression
ADR1-313 XhoI R	ATTACATGACTCGAGTCAACTGTTTCCCTTTAGATGATTTTCC	ADR1 overexpression
USV1-313 SpeI F	TTTTCTAGAACTAGTATGGAAAATACCACGAATCGTAATACTG	USV1 overexpression
USV1-313 XhoI R	ATTACATGACTCGAGTCAAGTCAATATGTAATCAACACTAAGCC	USV1 overexpression
CAT8-313 SpeI F	TTTTCTAGAACTAGTATGGCAAATAATAATTCTGATCGACAAG	CAT8 overexpression
CAT8-313 XhoI R	ATTACATGACTCGAGTTATTTGGCGTTTTGCCATTGG	CAT8 overexpression
XBP1-313 SpeI F	TTTTCTAGAACTAGTATGAAATATCCCGCTTTTAGCATTAACAG	XBP1 overexpression
XBP1-313 XhoI R	ATTACATGACTCGAGTTATTGTTTTGAGTTTGTTTTAAATTTGAAATTG	XBP1 overexpression
SUT1-313 SpeI F	TTTTCTAGAACTAGTATGTCCACAAGCATTACAGTAAGAAATAGAG	SUT1 overexpression
SUT1-313 XhoI R	ATTACATGACTCGAGCTAAAAATCAATGCTTTTATAGTCATCATAGG	SUT1 overexpression
Hap4-RF-423 SpeI F	GCAATCTAATCTAAGTTTCTAGAACTAGTATGACCGCAAAGACTTTTCTACTAC	HAP4 overexpression
Hap4-RF-423 XhoI R	AGCGTGACATAACTAATTACATGACTCGAGTCAAAATACTTGTACCTTTAAAAAATCG	HAP4 overexpression
Primers for deletion HAP4 or SUT1		
HAP4-D-F	ATTTGTTTTACCTACATTTTCTAGTACAAAAAAAAAACAAAAAAAGAATCTAGGTCTAGAGATCTGTTTAGCTTGC	
HAP4-D-R	TTTGTTTTCGTGATTTTTAGTTGTTTTCGTTTTATTGCAACATGCCTATTATTAAGGGTTCTCGAGAGCTCG	
SUT1-D-F	AAGTGCAACACATAATCTAAATCATTGAGGTCATCGTATGTCAAAAGAAGTAGGTCTAGAGATCTGTTTAGCTTGC	
SUT1-D-R	TTAATGCTAAATGCAAGTTTTGTGATGCTATTCATTAGAGATGCCTTTGTATTAAGGGTTCTCGAGAGCTCG	
KanB	CTGCAGCGAGGAGCCGTAAT	
HAP4-Vf	CCTTCACCTCTCTAAACCCCAG	
SUT1-Vf	CATACATGACAGATCCACATTTGC	
HAP4-Vf-R	CGGATATGTGAAAATGCTCTTAGG	
SUT1-Vf-R	AGGACTGTTCAAGCAATTCAATG	
